# Quantifying Exposure and Intra-Individual Reliability of High-Speed and Sprint Running During Sided-Games Training in Soccer Players: A Systematic Review and Meta-analysis

**DOI:** 10.1007/s40279-022-01773-1

**Published:** 2022-11-04

**Authors:** Antonio Dello Iacono, Shaun J. McLaren, Tom W. Macpherson, Marco Beato, Matthew Weston, Viswanath B. Unnithan, Tzlil Shushan

**Affiliations:** 1 Institute for Clinical Exercise and Health Science, School of Health and Life Sciences, University of the West of Scotland, Hamilton, UK; 2 Newcastle Falcons Rugby Club, Newcastle upon Tyne, UK; 3 Department of Sport and Exercise Sciences, Durham University, Durham, UK; 4 School of Health and Sports Sciences, University of Suffolk, Ipswich, UK; 5 Institute for Sport, Physical Education and Health Science, Moray House School of Education and Sport, University of Edinburgh, Edinburgh, UK; 6 School of Health Sciences, Western Sydney University, Sydney, NSW Australia

## Abstract

**Background:**

Sided games (i.e., small sided, medium sided, large sided) involve tactical, technical, physical, and psychological elements and are commonly implemented in soccer training. Although soccer sided-games research is plentiful, a meta-analytical synthesis of external load exposure during sided games is lacking.

**Objective:**

The objective of this systematic review and meta-analysis was to: (1) synthesize the evidence on high-speed and sprint running exposure induced by sided games in adult soccer players, (2) establish pooled estimates and intra-individual reliability for high-speed and sprint running exposure, and (3) explore the moderating effects of game format and playing constraints.

**Methods:**

A literature search was conducted in accordance with the Preferred Reporting Items for Systematic Reviews and Meta-Analyses 2020 guidelines. Four databases (PubMed/MEDLINE, Scopus, SPORTDiscus, Web of Science Core Collection) were systematically searched up to 25 January, 2022. Eligibility criteria were adult soccer players (population); training programs incorporating sided games (intervention); game manipulations including number of players, pitch dimension, and game orientation (comparator); and high-speed, very high-speed, and sprint relative (m$\cdot$min^−1^) running distances and associated intra-individual reliability (outcome). Eligible study risk of bias was evaluated using RoBANS. Pooled estimates for high-speed and sprint running exposure, and their intra-individual reliability, along with the moderating effect of tracking device running velocity thresholds, pitch dimension (i.e., area per player), and game orientation (i.e. score or possession), were determined via a multi-level mixed-effects meta-analysis. Estimate uncertainty is presented as 95% compatibility intervals (CIs) with the likely range of relative distances in similar future studies determined via 95% prediction intervals.

**Results:**

A total of 104 and 7 studies met our eligibility criteria for the main and reliability analyses, respectively. The range of relative distances covered across small-sided games, medium-sided games, and large-sided games was 14.8 m$\cdot$min^−1^ (95% CI 12.3–17.4) to 17.2 m$\cdot$min^−1^ (95% CI 13.5–20.8) for high-speed running, 2.7 m$\cdot$min^−1^ (95% CI 1.8–3.5) to 3.6 m$\cdot$min^−1^ (95% CI 2.3–4.8) for very high-speed running, and 0.2 m$\cdot$min^−1^ (95% CI 0.1–0.4) to 0.7 m$\cdot$min^−1^ (95% CI 0.5–0.9) for sprinting. Across different game formats, 95% prediction intervals showed future exposure for high-speed, very high-speed running, and sprinting to be 0–46.5 m$\cdot$min^−1^, 0–14.2 m$\cdot$min^−1^, and 0–2.6 m$\cdot$min^−1^, respectively. High-speed, very high-speed running, and sprinting showed poor reliability with a pooled coefficient of variation of 22.8% with distances being moderated by device speed thresholds, pitch dimension, and game orientation.

**Conclusions:**

This review is the first to provide a detailed synthesis of exposure and intra-individual reliability of high-speed and sprint running during soccer sided games. Our estimates, along with the moderating influence of common programming variables such as velocity thresholds, area per player, and game orientation should be considered for informed planning of small-sided games, medium-sided games, and large-sided games soccer training.

**Clinical Trial Registration:**

Open Science Framework available through https://osf.io/a4xr2/.

## Key Points

**Table:** 

In view of the extensive use of sided-games training in soccer, we synthesized the evidence on high-speed and sprint running exposure induced by sided games in adult soccer players, established pooled estimates and the associated intra-individual reliability for these external training load measures, and explored the moderating effects of a sided-game format and playing constraints.
Relative high-speed, very high-speed, and sprint running exposure induced by sided games, irrespective of format, are not comparable to the corresponding outcomes reported for regular 11-a-side soccer matches.
High-speed external load measures are highly variable, irrespective of a sided-game format.
We provide robust evidence for coaches and practitioners when manipulating playing constraints such as the relative area per player, the game orientation, and the pitch length-to-width ratio, and calibrating the velocity thresholds of tracking devices to predict high-speed, very high-speed, and sprint running exposure expected from sided-games training.
To help users intuitively visualize the findings of the meta-analytical and meta-regression models as well as to predict expected high-speed, very high-speed, and sprint running exposure scenarios upon planning soccer sided-games training, we developed a web application called “Sided-games Training App”.

## Introduction

Sided games have been part of the soccer coaching lexicon since the 1960s with the early documented publications describing their use for coaching the principles of play through mimicking technical and tactical soccer-playing scenarios [1, 2]. In the last two decades, sided games are a prevalent training method implemented by soccer coaches and practitioners [3], and they are widely adopted as game-based coaching pedagogical approaches in many worldwide talent developmental programs [4–6]. This widespread use of sided games in applied settings has attracted interest among sport scientists and researchers resulting in an exponential proliferation of research examining sided-games construct validity [7–13] through the associated physiological responses [7, 8, 14, 15] as well as defining evidence-based methodological recommendations for appropriate prescription and implementation [3, 9, 14, 16–18].

Sided games are modified games of short durations (e.g., 2–5 sets $\times$ 2–10 min) played on reduced pitch areas (e.g., 15 $\times$ 10 m^2^ up to 90 $\times$ 60 m^2^), often using adapted rules (e.g., scoring methods, permitted actions, specific tactical instructions) and involving fewer players (e.g., 2 vs 2 up to 10 vs 10 with or without goalkeepers) than traditional soccer match play [15, 16]. Conceptually, the foremost rationales for the use of sided games are specificity and efficiency [19], as the multi-dimensional demands of competitive soccer match (i.e., technical skills [20–22], tactical instructions [17, 23–25] and physical performance [18, 21, 23, 26]) can be replicated selectively or concurrently via bespoke game format configurations. Accordingly, in the soccer scientific literature, sided games are referred to as skill-based, game-based, or conditioning-based training depending on whether coaching prioritizes technical, tactical, or physical development, respectively [12, 22, 27]. Sided games are an integrated training method deemed to concurrently target several training goals such as: (1) to induce acute physiological responses (i.e., heart rate and maximal oxygen consumption) of comparable or greater intensity than matches [7, 8, 15, 25, 26, 28], which accumulating over time may induce positive fitness adaptations [9, 14]; (2) to replicate tactical behaviors of competitive match play while requiring players to make decisions and execute technical actions under ecological contextual constraints (e.g., opponents and fatigue) [4, 12, 17, 23, 24]; (3) to mimic the intermittent activity profile and physical demands (i.e., external load traits) of a soccer match whereby transfer effects on surrogate measures (e.g., accelerations, decelerations, sprints, and changes of direction) of soccer-specific performance are expected [18, 21, 23, 25, 26, 29, 30]; and (4) to increase player engagement and motivation due to ball integration [31–34]. Furthermore, sided games are also promoted as a holistic talent identification tool to discriminate between more and less talented youth players. In particular, players rated as more talented by their coaches are also more successful during sided games regardless of their team combinations and capable of covering a greater distance and playing at a higher speed compared with less talented peers. Thus, standard sided-games formats have the potential to be used to identify individuals with the capability to perform more successfully at the 11-a-side level [35–37].

While sided games constitute a specific training solution in soccer, their eligibility as a “One Size Fits All” method has been recently questioned by assumptions pointing to some practical limitations worthy of consideration [38, 39]. For example, the physical responses to sided games are influenced by many training variables such as the format and volume (e.g., number of games, duration, and rest intervals) or the technical and tactical dimensions of sided games as well as the individual player characteristics (i.e., including sex, training background, and baseline fitness level or even other mental and psychological aspects) [40]. From a validity construct, the concept of specificity is the leading rationale justifying the use of sided-games training to replicate match demands and induce an overloading stimulus in a match-like approach. However, while the overall relative external load intensity (relative distance [m·min^−1^]) is comparable between sided games and matches, studies investigating high-speed and sprint running distances between sided games and official matches do not support this validity assumption as the high-speed external load measures are largely disparate [41–44]. In this regard, high-speed and sprint running distances in official matches have considerably increased over the last 15 years (~ 29% increase and ~ 50% increase, respectively), and now represent ~ 7–11% and ~ 1–3% of the total distance covered during a match, respectively [45, 46]. Furthermore, high-speed and sprint activities are also considered as key determinants for successful outcomes during scoring situations [47–49]. Finally, the intra-individual variability of high-speed and sprint exposure to sided games is yet to be adequately elucidated.

In a recent systematic review [50], Clemente and colleagues collected longitudinal studies reporting reliability data and those purposefully designed to investigate the reliability of load outcomes observed during sided games. The authors highlighted poor inter-individual reliability especially for high-speed running and sprint distances [11, 29, 39, 42, 51–56]. This evidence is an important step in the right direction as it summarizes the inter-individual variability of training load measures during sided games. However, the authors neither established pooled estimates of the inter-individual reliability scores nor, and more importantly, provided any insights on the intra-individual reliability of high-speed and sprint running distances. Given that a variety of sided-games formats are regularly used in training, comprehensive knowledge of their effect on high-speed and sprint running exposure, as well as the intra-individual reliability of these measures, would appear paramount for a thorough and informed prescription of individual internal and external training loads and for the subsequent evaluation and planning of the training effects.

The evidence on sided games in soccer is noticeably extensive as recently confirmed in an umbrella review encapsulating the systematic reviews and meta-analyses performed on this topic [3]. Here, authors reported the findings of eight systematic reviews and two meta-analyses [4, 15, 16, 30, 57–60] summarizing the short-term and long-term effects of sided-games on a variety of physiological, physical, and psychological characteristics as well as technical-tactical dimensions. The available literature on sided games in soccer and the recent contribution of Clemente et al. [3] are certainly relevant to guide the planning, design, and implementation of sided games among soccer coaches and practitioners. However, a critical revision of the same literature uncovers three key aspects that warrant further consideration: (1) external load measures of high-speed and sprint running exposure for different sided-games formats were reported only in one systematic review [30] from the eight synthesized by Clemente et al. [3]; (2) a meta-analytical synthesis of the pooled estimates pertaining to these external load metrics has yet to be performed, and (3) the intra-individual variability in response to sided games is underdetermined. Knowledge on these aspects holds a potential practical impact, with the anticipated evidence readily informing implementation of sided-games training in applied settings as well as likely guiding future directions in soccer research. A rigorous synthesis of the current sided-games literature is therefore warranted.

Accordingly, the aims of this systematic review and meta-analysis were to synthesize the existing evidence on high-speed and sprint running exposure induced by sided games in adult soccer players, and to establish pooled estimates for these external training load measures as well as the associated intra-individual reliability, while exploring the moderating effects of sided-games formats and playing constraints. Importantly, our review is confined to high-speed and sprint running exposure, not the effectiveness of sided-games training as a fitness intervention.

## Methods

### Searching Strategy

This systematic review and meta-analysis were conducted in accordance with the Preferred Reporting Items for Systematic Reviews and Meta-Analyses (PRISMA) 2020 guidelines [61, 62] (items checklist available in the Electronic Supplementary Material [ESM]), alongside the consensus statement for reviews in Exercise, Rehabilitation, Sport medicine and SporTs science (PERSiST) [63], and was registered [64] in the Open Science Framework (https://osf.io/gh792) on 4 March, 2021. Two reviewers (ADI, TS) and a senior librarian with ~ 15 years of experience in conducting systematic searches for meta-analyses in sport performance fields independently performed standard and optimized electronic searches using the PubMed/MEDLINE, Scopus, SPORTDiscus and Web of Science Core Collection databases, from inception to 28 April, 2021 (further details in the ESM: https://osf.io/28vap).

The research questions were defined by the PICOS approach:

- *Population* male and female football/soccer players with aged 17 years or older.
- *Intervention* sided games performed as part of regular soccer training, irrespective of training intervention duration.
- *Comparator* sided-games format characteristics of number of players, pitch dimension, and inclusion or not of goalkeepers.
- *Outcomes* external load metrics of high-speed, very high-speed, and sprint running distances exposure and associated intra-individual reliability scores.
- *Study design* any quantitative research design that met the above criteria.

The search criteria and strategy were based on authorship expertise and familiarity with soccer sided-games terminology. Relevant keywords for each search term were determined through pilot searching (screening of titles, abstracts, keywords, and full texts of previously known studies). An overview of the search strategy is presented in Table 1. Additionally, we screened the reference lists of included studies, contacted experts in the field (e.g., authors of included studies), and regularly searched for information on additional trials, including unpublished or ongoing studies through the ResearchGate network (http://www.researchgate.net) and Twitter websites (http://www.twitter.com). All searches were finally updated on 25 January, 2022. On the same date, we also screened for any correction notice, expression of concern, retraction, and removal pertaining to the final pool of studies included in the meta-analysis to ensure the integrity of the scholarly record and the accuracy of the data.

**Table 1:** Searching strategy

Level 1	Football OR soccer OR "football player*" OR "football athlete*" OR "soccer player*" OR "soccer athlete*"
Level 2	"Small-sided games" OR SSG OR SSGs OR "game-based training" OR "condition* game" OR "condition* drill" OR "standard* drill" OR "standard* game" OR "position* game" OR "position* drill"
Level 3	"External load*" OR "external training load*" OR "external TL" OR "physical demand*" OR performance OR "run* performance" OR "physical performance" OR activ* OR intens* OR "movement pattern" OR "time-motion" OR "activ* profile" OR "high-speed run*" OR "high-speed distance" OR "high-intensity run*" OR "high-intensity distance*" OR sprint*

Level 1 AND Level 2 AND Level 3. Note: For the systematic search of eligible articles, two complementary searching strategies were used. The first, as detailed above, was based on a traditional searching process implementing keywords merged within and between levels by using the Boolean operators OR and AND, respectively. These operators were applied to ALL FIELD in PubMed/MEDLINE, Web of Science, and SPORTDiscus and to Article title, Abstract, and Keyword in Scopus. Moreover, no filters or automatic tools were used for the searching process. Then, following consultation with a senior librarian, an expert in designing searching strategies for systematic review and meta-analysis articles, the searching strategy was optimized using proximity operators to merge keywords across different levels. Briefly, the operator W/3 was used in Scopus and SPORTDiscus bibliographic databases, while the operator near/3 was used to replicate the search in the Web of Science bibliographic database. Finally, a similar optimized strategy was performed in PubMed/MEDLINE by adding the use of search filters containing Medical Subject Headings (MeSH). Full details of the two complementary searching strategies are available in the ESM

### Screening Strategy and Study Selection

Two reviewers (ADI, TS) assessed relevant records, which were downloaded into Endnote (version 20; Clarivate Analytics, Philadelphia, PA, USA) and then to a Microsoft^®^ Excel spreadsheet (Microsoft, Redmond, WA, USA). Duplicate records were identified and removed, and an assessment of the remaining studies was undertaken sequentially (i.e., criteria 1–7) according to the inclusion–exclusion criteria described in Table 2. Regarding inclusion criteria 4 (i.e., age of the participants), we decided to include players aged 17 years and older although from a chronological age perspective they may not be considered adult. However, at this age they are clearly post-peak height velocity, and consequently, biological maturity status is not a confounding factor for any of the outcome measures [65, 66]. Based on the other criteria, more studies were discarded, and full-text studies finally retrieved and assessed independently by both reviewers for inclusion scrutiny.

**Table 2:** Inclusion–exclusion criteria of studies to be included in the systematic review and meta-analysis

Criteria	Inclusion	Exclusion
1^a^	Relevant sided-games topic (e.g., format characteristics, number of players, pitch dimension, inclusion or not of goalkeepers)	Studies reporting combined training and match data
2	Original research study	Reviews, surveys, opinion pieces, books, periodicals, editorials, case studies, non-academic/non-peer-reviewed text
3	Full text available in English	No access to full text in English
4^a^	Participants are soccer players of any sex and with an age of 17 years or older	Youth players (aged younger than 17 years) or non-soccer players (e.g., other team sports)
5^a^	Observational studies	Intervention studies with a single group for pre-post intervention comparisons where training responses could not be extrapolated
6^a^	High-speed running, very high-speed running, and sprint distances outcomes defined according to fixed-speed thresholds and collected with tracking systems (e.g., global navigation satellite system, local positioning system, camera-based system)	High-speed running and sprint distances outcomes not collected or defined according to individual thresholds not clearly reported, or not attainable
7^a^	Reliability data (e.g., intra-individual technical error of measurement or coefficient of variation) reported, calculated from the descriptive data reported in the manuscript, or calculated from raw data provided by authors following contact	Reliability data not accessible or attainable

^a^Criteria defined by the PICOS approach

### Data Extraction and Coding

Two reviewers (ADI, TS) independently extracted data using a dedicated form (see ESM: https://osf.io/4jbhg). Independent screening results were then combined, and any disagreements were resolved by consensus discussion (*n* = 6). For studies meeting the final inclusion criteria, the following data were extracted: (1) bibliographic information, (2) player characteristics: sample size, sex, age, and competitive level; (3) sided-games characteristics: format, dimensions (length × width), length:width ratio (AU), area per player (m^2^), configuration (sets × duration [min]), recovery between sets (min), game orientation, presence of coach encouragement and number of touches (*n*); (4) load monitoring technology details: model, sampling frequency (Hz), velocity category and respective thresholds; and (5) summary statistics included in the meta-analysis.

As a means of data reduction and to facilitate the meta-analytical and meta-regression analyses, the following decisions were made in line with the literature on soccer sided games [3, 67–69] as well as upon reaching consensus between the authors of this review. To illustrate, the sided-games formats were grouped based on the number of players in:

- small-sided games (SSG): 2v2 to 4v4;
- medium-sided games (MSG): 5v5 to 7v7;
- large-sided games (LSG): 8v8 to 10v10.

This categorization was made considering only the number of outfield players (i.e., excluding the goalkeepers). Unbalanced game formats (i.e., different number of players per team) were coded as follows:

- If the additional players moved only outside of the playing area (e.g., bouncers and floaters), the sided game was coded based on the number of outfield players regardless of the number of additional players (e.g., 4v4 + 1/2/3/4 bouncers/floaters $\to$ 4v4).
- If the additional players actively took part in the game and were allowed to move within the playing area (e.g., jollies and wildcards), then two further criteria were applied:
  - When the numerical advantage provided by the additional players was $\le$ 50%, the sided game was coded based on the number of outfield players (e.g., 4v4 + 2 jollies and 6v6 + 3 jollies $\to$ 4v4 and 6v6, respectively).
  - When the numerical advantage provided by the additional players exceeded 50%, the sided game was discarded and not included in the meta-analysis (e.g., 4v4 + 3 jollies and 6v6 + 4 jollies $\to$ no format), as it was considered as a tactical drill rather than a sided game.

The relative areas per player were recalculated for studies where goalkeepers were not considered in the original calculation. Accordingly, areas per player were adjusted for the total number of players and reflected the effective relative playing areas. Considering the game orientation variable, formats were coded either as score oriented or possession oriented if they included or did not include goalkeepers or mini goals, respectively. Regarding the summary statistics, we calculated “overall exposure” measures as the aggregated distances across the external load outcomes from the same sample, with a minimum velocity threshold corresponding to the lower bound of the high-speed running band and a maximum velocity threshold set at infinite (*Note*: four studies had a fixed maximum velocity threshold rather than an infinite value) as to include any distance above the sprint distance threshold. To this end, we calculated the mean of the overall exposure measures as the arithmetic sum of the means of the different external load outcomes (i.e., *x̄*_1_ + *x̄*_2_ and *x̄*_1_ + *x̄*_2_ + *x̄*_3_ when aggregating two or three external load outcomes, respectively). The aggregated standard deviation (σ_agg_) was calculated according to the variance sum law for dependent variables [70]. We provide a comprehensive description of the procedural steps of this approach in the ESM (https://osf.io/vsr4d). Intra-individual reliability was expressed as a relative measure of reliability (i.e., coefficient of variation [CV; %]) and calculated according to Hopkins [71]. Effect sizes were log-transformed and adjusted for sample size [72, 73], and subsequently back-transformed (including the bias correction for sample size) for analysis interpretations of the pooled estimates.

### Handling Missing and Duplicate Data

To handle missing data and attain missing information, we used direct contact details of the first or corresponding author(s) along with their social network accounts (e.g., ResearchGate, Twitter). To clarify, one author (ADI) e-mailed the first or corresponding author(s) of the study requesting the raw data or mean and standard deviation values. If the authors did not respond to the first e-mail, a reminder was sent after 2 weeks. If the authors did not reply within 1 month from the remainder e-mail, we calculated the outcomes based on the figures (i.e., data were digitized using WebPlotDigitizer; version 4.3, Ankit Rohatgi; https://apps.automeris.io/wpd/) and tables. Where mean (*n* = 2) and standard deviation (*n* = 4) data were not provided by authors nor could be extracted based on figures, we handled missing values by a calculation according to the methods and customized Microsoft^®^ Excel spreadsheet (Microsoft, Redmond, WA, USA) calculators suggested by Hozo et al. [74] and Wan et al. [75], respectively. Prior to proceeding with the data analysis and following an inspection of the full dataset, four studies were found to report the same data for the same estimates in different publications of the same author(s). Therefore, the duplicate data were removed, and single records were used for the analysis.

### Data Analysis

#### Overall Meta-analysis

Data analyses were conducted using the ‘metafor’ [76] and ‘clubSandwich’ [77] packages for R studio environment (version 1.4.1106) [78]. All analysis codes are presented in the ESM (https://osf.io/28wku, https://osf.io/fywv8). In most of the included studies, we were able to extract more than a single-effect size. Multiple-effect sizes were within studies and derived from a variety of sided-games characteristics, including game format (e.g., number of players, unbalanced teams), game configuration (number of sets, set duration, recovery between sets), pitch dimensions and orientation (e.g., area per players, length:width ratio), game objectives (score-oriented vs possession), and other rule modifications (number of touches, offside rule).

Given the hierarchical structure in our datasets (i.e., multiple-effect estimates nested within clusters), as well as the likelihood of statistical dependency, we employed a recently developed approach using a multi-level mixed-effects meta-analysis and robust variance estimation [79]. Such an approach allows exploration of the heterogeneity present across multiple levels, hence, within-group and between-group variance [80], and provides a robust method for the meta-analysis results while accounting for the dependency of effect estimates derived from common samples [81]. In such cases, it has been proposed to account for the correlation between effect estimates by replacing their sampling variance with the entire ‘V matrix,’ indicating the variance–covariance matrix of the estimates [79, 82]. As it was not possible to attain the correlation between effect estimates drawn from the same participants in most of the included studies, we reanalyzed previous data of our research group and external collaborators (*n* = 85), which yielded an assumed constant correlation of 0.5. In the ESM (https://osf.io/fywv8), we report sensitivity analyses whereby a range of correlation values were used to evaluate the influence of the changes in the within-group covariance on the pooled estimates and its variance components. Collectively, these analyses showed identical pooled estimates and nearly similar variance components (see ESM: https://osf.io/pdj37, https://osf.io/z2qjg).

For effects emerging from the main results and meta-regression analyses, we opted to avoid a dichotomous approach for their interpretation based upon traditional null hypothesis significance testing, which has been extensively criticized [83, 84]. Alternatively, we considered the practical implications of all results with an emphasis on the pooled point estimates as well as the lower and upper limits of the interval estimates [64]. Uncertainty in meta-analysis estimates was expressed using 95% compatibility (confidence) intervals (CIs), representing ranges of values compatible with our models and assumptions. We also derived 95% prediction intervals (PIs), which convey the likely range of the true measurement properties in similar future studies [64].

#### Heterogeneity and Moderating Effects

To describe the extent of heterogeneity, we calculated Q-statistics, as well as restricted maximum likelihood estimates of the within-group (*τ*_2_) and between-group (*τ*_3_) variances (standard deviation; tau [*τ*]) [85], and the *I*^2^ of the within-group ($I_{2}^{2}$) and between-group ($I_{3}^{2}$) variances [86]. The *I*^2^ implies the percentage of variance that is due to study heterogeneity rather than sampling error [86]. Of note, because many studies reported effect sizes equal to 0 (mean and standard deviation = 0 m), neither Q nor *I*^2^ statistics could be computed for these models, and in these cases we reported the *τ*-statistic only. To examine possible sources of heterogeneity and moderating effects, we conducted meta-regression analyses with four variables from the format and monitoring characteristics, including three continuous moderators (velocity thresholds, area per player, and length:width ratio), with game orientation (i.e., possession vs score) as a categorical moderator. For the continuous moderators, their effects were interpreted as the changes associated with pre-defined values from fixed anchor references as follows:

- *Velocity thresholds* the effects associated with $\pm$ 1 km $\cdot$ h^−1^ change of the velocity thresholds set in the monitoring devices from the anchored fixed references of 14.4, 19.8, and 22.0 km $\cdot$ h^−1^ for high-speed, very high-speed, and sprint, respectively (i.e., approximately middle value of the ranges found in the literature for each speed zone).
- *Area per player* the effects associated with an increase/decrease of 25 m^2^ of the relative area per player from the anchored fixed reference of 100 m^2^ per player.
- *Length:width ratio* the effects associated with $\pm$ 0.2 AU change of the length:width ratio from the anchored fixed reference of 1 AU (i.e., equal length and width dimensions).
- *Game orientation* this was examined by comparing score-oriented and possession-oriented formats with the possession-oriented category used as the reference.

### Risk of Bias

For the systematic review of the external load outcomes and associated reliability measures, eligible study risk of bias was evaluated using Risk of Bias Assessment Tool for Non-randomized Studies (RoBANS) [87]. This comprehensive framework assesses six different bias domains including: participant selection, confounding variables, exposure measurement, outcome assessments blinding, incomplete outcome data, and selective outcome reporting (ESM; https://osf.io/vczdg). The RoBANS was assessed by two authors (ADI, TWM), and a third author (TS) acted as a moderator if there were discrepancies in the interpretation of the risk of bias assessment.

### Small-Study Effect Bias

All datasets included the minimum number (ten studies) required for formal testing of asymmetry [88]. Small-study effects were visually inspected using funnel plots [89]. To confirm our visual impression, Egger’s regression test (by fitting the square root of the sampling variance as a moderator) was employed [90].

## Results

### Search Results

**Fig. 1:**
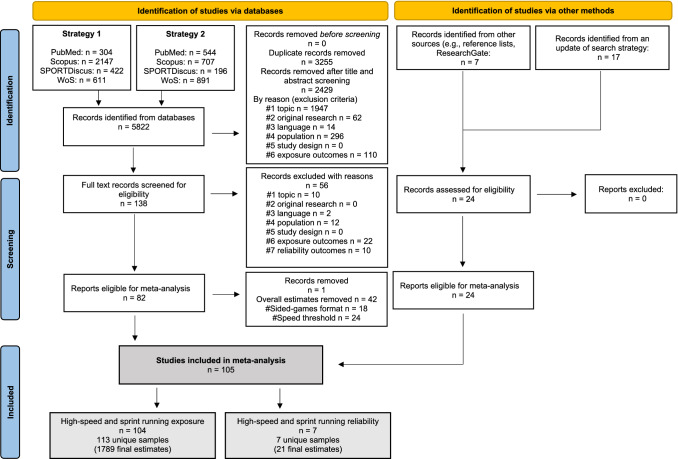
Preferred Reporting Items for Systematic Reviews and Meta-Analyses (PRISMA) flowchart. *﻿WoS* Web of Science

The search and screening process is presented in the PRISMA flow chart (Fig. 1). The initial search identified 5822 relevant studies, with 2567 remaining after the removal of duplicates (*n* = 3255). An additional 2429 studies were excluded following title and abstract screening, and 138 full-text studies were then assessed for eligibility. Based on our inclusion criteria, a total of 82 studies were selected and 56 were excluded due to: not written in English (*n* = 2, [91, 92]), not complying with the population criteria (*n* = 12, [18, 23, 93–101]), intervention criteria (*n* = 10, [102–111]), and outcomes criteria (exposure outcomes: *n* = 22; reliability outcomes: *n* = 10, [22, 41, 44, 109, 111–138]) [see Fig. 1 “Records excluded with reasons”). We discarded one study (*n* = 4 estimates) [139] and other estimates where sided-games formats (*n* = 14) could not be coded, or when the velocity thresholds (*n* = 24) were not calculated according to our defined ranges.

An additional 24 studies were identified from the updated searching round and other sources, resulting in 105 studies meeting the inclusion criteria. One study [133] was included in the intra-individual reliability analysis only because of not reporting descriptive data of exposure. Accordingly, the final dataset for high-speed and sprint running exposure included 104 studies (113 samples and 1789 estimates), with 188, 247, and 213 estimates used to examine high-speed running in SSG, MSG, and LSG, respectively; 226, 238, and 194 estimates used to examine very high-speed running in SSG, MSG, and LSG, respectively; and 103, 177, and 203 used to examine sprint running in SSG, MSG, and LSG, respectively. Seven independent studies (7 samples and 21 CV estimates) were included in the meta-analysis of the intra-individual reliability of the same external load measures. Full details of all included studies can be seen in the data extraction table (ESM; https://osf.io/5hzve).

### Study Characteristics

Descriptive information for all 105 studies is displayed in Table 3. The pooled number of participants was 1962 with sample sizes that ranged from 6 to 62 participants (median *n* = 16) per group within each study. The total sample included 66 female and 1876 male players (sex not reported for *n* = 20) with a mean age range from 19.1 to 24.3 years and from 17 to 28.7 years, respectively. Of these, 227 players were aged between 17 and 18 years and there were 1735 adult players. The samples across all players were classified as Tier 2 (*n* = 600), Tier 3 (*n* = 1176), and Tier 4 (*n* = 130) [140], while the competitive level was not reported for the remaining players (*n* = 56). Most of the included studies (*n* = 96) used a global navigation positioning system (GNSS) or GNSS combined with micro-electromechanical system technology to collect external load outcomes. In four studies, the external load outcomes were collected using either optical (*n* = 1) or local position measurement technologies (*n* = 3). In the five remaining studies, technology was not reported. In more than half of the studies (*n* = 53), sampling frequency of the tracking technology was 10 Hz, with the remaining studies reporting sampling frequencies of 1 Hz (*n* = 1), 5 Hz (*n* = 21), 15 Hz (*n* = 10), 18 Hz (*n* = 4), 20 Hz (*n* = 3), 24 Hz (*n* = 1), 40 Hz (*n* = 1), and 42 Hz (*n* = 1). In ten studies, sampling frequency was not reported. The most common thresholds used to define high-speed (*n* = 24), very high-speed (*n* = 27), and sprint (*n* = 11) running distances were 13 km $\cdot$ h^−1^ (range: 12.2–18 km $\cdot$ h^−1^), 19.8 km $\cdot$ h^−1^ (range: 16–21.6 km $\cdot$ h^−1^), and 25.2 km $\cdot$ h^−1^ (range: 18–25.2 km $\cdot$ h^−1^), respectively. The number of satellites used to infer GNSS signal quality was reported in four studies [56, 141–143], ranging from 3 to 20. Horizontal dilution of precision used to indicate the accuracy of the GNSS horizontal positional signal was reported in three studies [141–143] and was 0.54 $\pm$ 0.20.

**Table 3:** Summary of data extracted from the included studies

Study	Athletes	Sided games characteristics										Device specification		Velocity threshold (km $\cdot$ h^−^1)		
	*N* Sex Age (years) Level	Format	Additional players	Dimension (m × m)	L:W Ratio	ApP (m^2^)	Configuration (games × min)	Recovery (min)	Score orientation	Coach encouragement	Touches	Technology (model)	Sampling frequency (Hz)	HSR	VHSR	SR
Aasgaard and Kilding [144]	8 Male 23.6 Tier 2	2v2 3v3 4v4	No	20 × 20 30 × 21 33 × 26	1 1.4 1.3	100 105 108.1	4 × 4	6	Yes	Yes	Free	GNSS + MEMS (VX Sport 220, Visuallex Sport International)	10 + 100	13.1	17.9	21
Ade et al. [55]	16 Male 17 English Tier 3	2v2	No	27 × 18	1.5	121.5	8 × 1	1	Yes	Yes	Free	GNSS + MEMS (MinimaxX, version 4.0, Catapult)	10 + 100	14.4	19.9	25.2
Aguiar et al. [25]	10 Male 18 Portuguese Tier 3	2v2 + GK 3v3 + GK 4v4 + GK 5v5 + GK	No	NA	1.3	100 112.5 120 125	3 × 6	5	Yes	No	Free	GNSS (SPI Elite System GPSports)	5	13	16	18
Aquino et al. [145]	8 Male 17 Brazilian Tier 3 8 Male 20 Brazilian Tier 3	6v6 + GK	No	49 × 25	2	87.5	6 × 4	7.5	Yes	Yes	Free	GNSS (MinimaxX, Catapult)	5	17 18		
Asian-Clemente et al. [146]	15 Male 24.4 Spanish Tier 2	3v3 3v3 + 2 5v5 5v5 + 2 7v7 7v7 + 2	F	30 × 20 30 × 20 37 × 27 37 × 27 44 × 32 44 × 32	1.5 1.5 1.37 1.37 1.37 1.37	100 75 136.9 114 101 88	4 × 4	2	No	Yes	NA	GNSS (SPI Elite System GPSports)	5	14	18	
Avalos-Guillen et al. [147]	17 Male 24 Costa Rican Tier 3	5v5 + GK	No	32 × 32	1	85.3	1 × 8	No	Yes	NA	Free	GNSS (SPI Pro II, GPSports)	15	14.1	19.1	
Batista et al. [148]	16 Male 23.9 Tier 2	7v7 + GK	No	62 × 50	1.2	187.5	2 × 5	3	Yes	No	Free	GNSS (NA)	5	14.4	19.8	
Baptista et al. [149]	23 Male 24.9 Tier 2	7v7 + GK	No	62 × 50	1.2	187.5	2 × 5	3	Yes	No	Free	GNSS (NA)	5	14.1	19.8	
Brandes and Elvers, [150]	16 Male 17.2 German Tier 3	4v4 + GK	No	40 × 40	1	160	3 × 4	4	Yes	Yes	Free	GNSS (Qstartz, Q1000Ex)	5	13	18	21
Branquinho et al. [151]	20 Male 25.2 Portuguese Tier 3	4v4 + GK	No	40 × 40	1	160	1 × 24 2 × 12 4 × 6 6 × 4	No 2	Yes	NA	Free	GNSS (Wimu Pro, Realtrack Systems)	10	14.4	19.8	
Branquinho et al. [152]	16 Male 23.9 Tier 2	4v4 + GK	No	40 × 40	1	160	1 × 18 3 × 6	0.5, 1, 1.5, 2	Yes	NA	NA	GNSS (Wimu Pro, Realtrack Systems)	10	19	22	24
Bujalance-Moreno et al. [153]	16 Male 23.9 Tier 2	4v4	No	30 × 20	1.5	75	4 × 4	2	No	NA	Free	GNSS (Wimu Pro, Realtrack Systems)	10	13	18	
Bujalance-Moreno et al. [154]	16 Male 23.9 Tier 2	4v4 4v4 + 2	No J	30 × 20	1.5	75 60	4 × 4	2	No	NA	Free	GNSS (Wimu Pro, Realtrack Systems)	20	13	18	24.1
Bujalance-Moreno et al. [155]	16 Male 23.9 Tier 2	4v4	No	30 × 20	1.5	75	4 × 4	2	Yes and No	NA	Free	GNSS (Wimu Pro, Realtrack Systems)	20	13	18	24
Casamichana et al. [156]	10 Male 21.3 Spanish Tier 2	5v5	No	55 × 38	1.45	210	1 × 16 4 × 4 2 × 8	No 1 2	No	NA	Free	GNSS + MEMS (MinimaxX, v.4.0, Catapult)	10 + 100	13	18	21
Casamichana et al. [157]	12 Male 22.7 Tier 2	6v6	No	60 × 49	1.22	245	1 × 12	No	No	Yes	2&Free	GNSS + MEMS (MinimaxX, v.4.0, Catapult)	10 + 100	13	18	
Casamichana et al. [158]	18 Male 23.4 Tier 2	3v3 6v6 9v9	No	19 × 29 40 × 28 55 × 30	0.65 1.43 1.83	92 93 92	NA	NA	No	Yes	Free	GNSS + MEMS (MinimaxX, v.4.0, Catapult)	10 + 100	13	18	21
Casamichana et al. [159]	20 Male 21 Tier 2	5v5 + GK	No	25 × 40 25 × 66 50 × 40 50 × 66	0.62 0.38 1.25 0.75	83.3 137.5 167 275	4 × 6	8	Yes	Yes	Free	GNSS + MEMS (MinimaxX, v.4.0, Catapult)	10 + 100	13	18	21
Castellano et al. [160]	14 Male 21.3 Spanish Tier 2	3v3 3v3 + GK 5v5 5v5 + GK 7v7 7v7 + GK	No	43 × 30 43 × 30 55 × 38 55 × 38 64 × 46 64 × 46	1.43 1.43 1.45 1.45 1.39 1.39	215 161 209 174 210 184	3 × 6	5	Yes and No Yes Yes and No Yes Yes and No Yes	Yes	Free	GNSS + MEMS (MinimaxX, v.4.0, Catapult)	10 + 100	13	18	21
Castillo et al. [161]	16 Male 18.1 Youth Tier 3	4v4 + GK	No	30 × 20	1.5	60	4 × 4	3	Yes	No	Free	GNSS (Wimu Pro, Realtrack Systems)	10	14	21	
Castillo et al. [162]	14 Male 18.1 Spanish Tier 3	4v4 + GK	No	30 × 20	1.5	60	4 × 4	2	Yes	Yes	Free	GNSS (Wimu Pro, Realtrack Systems)	10	14		
Cihan [163]	18 Male 19.6 Youth Tier 3	2v2 + 2 3v3	J	20 × 35	0.57	117	4 × 3	3	No	Yes	Free	GNSS (GPSports, SPI)	NA	13	18	
Clemente et al. [164]	6 Male 20.3 Tier 2	3v3	No	19 × 24	0.79	76	3 × 3	3	Yes	Yes	Free	GNSS + MEMS (Johan Sports)	10 + 100	14	20	
Clemente [165]	10 Male 19.8 Youth Tier 2	5v5	No	42 × 22	1.9	92.4	6 × 3 3 × 6	2	Yes	Yes	Free	GNSS + MEMS (Johan Sports)	10 + 100	14.9	20	
Clemente et al. [166]	10 Male 23.7 Tier 2	5v5	No	42 × 22	1.9	92.4	3 × 6 6 × 3	2	Yes	Yes	Free	GNSS + MEMS (Johan Sports)	10 + 100	14	20	
Clemente et al. [167]	10 Male 19.8 Youth Tier 2	5v5	No	30 × 30	1	90	6 × 3 3 × 6	2	Yes	Yes	Free	GNSS + MEMS (Johan Sports)	10 + 100	14	20	
Clemente et al. [168]	22 Male 24.6 Portuguese Tier 3	5v5 + 2 5v5 + GK 10v10 + 2	F	40 × 31 52 × 44	1.29 1.18	103 104	2 × 6	3	No Yes No	Yes	Free	GNSS + MEMS (Johan Sports)	10 + 100	14	20	
Clemente et al. [52]	10 Male 18.3 Youth Tier 3	5v5	No	30 × 30	1	90	3 × 5	2	Yes	Yes	Free	GNSS + MEMS (Johan Sports)	10 + 100	14		
Clemente et al. [42]	23 Male 24.6 Portuguese Tier 3	5v5 + GK 6v6 + GK 9v9 + GK	No	40 × 31 45 × 32 70 × 50	1.29 1.4 1.4	103 175	2 × 6 3 × 7.5 2 × 11	3 3 6	Yes	Na	Free	GNSS + MEMS (Johan Sports)	10 + 100	14	20	
Clemente et al. [169]	10 Male 28.1 Portuguese Tier 3	5v5 + GK	No	60 × 30	2	150	3 × 4	2	Yes	Yes	Free	GNSS + MEMS (OptimeEye S5, Catapult)	10 + 100	14.4	19.8	
Coutinho et al. [170]	16 Male 17.9 NA	5v5 + GK	No	40 × 30	1.33	120	3 × 4	2	Yes	No	1, 2, free	GNSS (SPI Pro System GPSports)	5	14.4		
Dalen et al. [43]	18 Male 24.9 Norwegian Tier 3	4v4 + GK 6v6 + GK	No	39 × 39 47 × 43	1 1.1	152 144	6–8 × 3 3–5 × 6	3 2	Yes	NA	Na	Radiofrequency (ZXY Sport Tracking AS; ChyronHego Nasdaq)	20		19.8	25.2
Darbellay et al. [171]	14 Male 17 Switzer youth Tier 3	4v4 + GK 8v8 + GK	No	30 × 25 50 × 40	1.2 1.25	75 11	3 × 3 1 × 15	1.5 No	Yes	Yes	Free	GNSS (NA)	10	13	16	19
Dellal et al. [172]	20 Male 27.4 Ivory Coast Tier 4	2v2 + 4 3v3 + 4 4v4 + 4	F	20 × 15 25 × 18 30 × 20	1.3 1.4 1.5	75	4 × 2 4 × 3 4 × 4	2 3 4	No	Yes	1,2, free	GNSS (SPI Elite System GPSports)	5	13	18	
Dellal et al. [27]	20 Male 27.4 Tier 3	4v4 + 4	F	30 × 20	1.5	75	4 × 4	3	No	Yes	1, free	GNSS (SPI Elite System GPSports)	NA	13	18	
Dellal et al. [173]	20 Male 26.3 French Tier 2	2v2 + 4 3v3 + 4 4v4 + 4	F	20 × 15 25 × 18 30 × 20	1.3 1.4 1.5	75	4 × 2 4 × 3 4 × 4	3	No	Yes	1, 2, free	GNSS (SPI Elite System GPSports)	5	13	18	
Dellal et al. [174]	20 Male 27 Ivory Coast Tier 4	2v2 + 4 3v3 + 4 4v4 + 4	F	20 × 15 25 × 18 30 × 20	1.3 1.4 1.5	75	4 × 2 4 × 3 4 × 4	3	No	Yes	1	GNSS (SPI Elite System GPSports)	5	13	17	
Dello Iacono et al. [29]	20 Male 18.6 Israeli youth Tier 3	5v5 + GK	No	42 × 30	1.4	105	3–5 × 8	4–8	Yes	Yes	Free	GNSS (SPI Pro II, GPSports)	15		19	25.2
Falces-Prieto et al. [175]	12 Male 17.3 NA	4v4 + 4	J	20 × 20	1	33.3	3 × 4	3	No	NA	NA	GNSS + MEMS (Playertek, Catapult)	10 + 400		18	
Ferraz et al. [176]	20 Male 21.9 Portuguese Tier 2	5v5	No	40 × 20	2	80	2 × 10	1.5	No	Yes	Free	GNSS (SPI Elite System GPSports)	5	14.4	19.8	
Ferraz et al. [177]	20 Male 22.3 Portuguese Tier 3	5v5	No	40 × 20	2	80	2 × 10	1.5	No	Yes	Free	GNSS (SPI Elite System GPSports)	5	14.4	19.8	
Fransson et al. [178]	13 Male 21.1 Swedish Tier 3	6v6 + GK	No	40 × 32	1.25	91	2 × 7–9	2	Yes	Yes	Free	GNSS + MEMS (Catapult Sports)	10	14	21	
Gaudino et al. [179]	26 Male 26 English Tier 4	5v5 5v5 + GK 7v7 7v7 + GK 10v10	No	27 × 27 30 × 30 37 × 37 45 × 45 52 × 52	1	73 75 98 98 135	4	NA	No Yes No Yes No	NA	2	GNSS (SPI Pro X, GPSports)	15	14	19.8	25.2
Gaudino et al. [180]	26 Male 26 English Tier 4	5v5 + GK 7v7 + GK	No	30 × 30 45 × 35	1 1.3	75 98	1 × 5 1 × 8	No	Yes	Yes	2	GNSS (SPI Pro X, GPSports)	15	14		
Giménez and Gomez [181]	14 Male 23.2 Polish Tier 3	3v3 + GK 4v4	No	30 × 30 30 × 24	1 1.25	112 90	8 × 4	2	Yes	Yes	2	GNSS + MEMS (MinimaxX, v.4.0, Catapult)	10 + 100	15.1	18	
Giménez et al. [182]	14 Male 23.2 Polish Tier 3	3v3 + GK 7v7 + GK	No	30 × 30 42.4 × 42.4	1	112	8 × 4	2	Yes	Yes	2	GNSS + MEMS (MinimaxX, v.4.0, Catapult)	10 + 100	15.1	18	24.8
Giménez et al. [183]	14 Male 23.2 Polish Tier 3	4v4	No	30 × 24	1.25	90	3 × 4	3	No	Yes	1, 2, 3	GNSS + MEMS (MinimaxX, v.4.0, Catapult)	10 + 100	15	18	25
Gómez et al., [184]	25 Male 20.5 Spanish Tier 3	7v7 + 3 8v8 + 3	J	36 × 29 40 × 35	1.24 1.14	61.4 73.7	NA	NA	No	NA	Free	GNSS + MEMS (Viper Pod, STATSports)	10 + 100		19.8	
Goméz-Carmona [185]	16 Male 17.3 Youth Tier 2	6v6 6v6 + GK	No	25 × 40	0.62	83 71	2 × 5	5	No Yes	NA	Free	GNSS + MEMS (Wimu Pro, Realtrack Systems)	10 + 100	14	21	
Gonçalves et al. [186]	19 Male 25.1 Spanish Tier 3	10v9 + GK	No	58.5 × 64	0.91	178	2 × 5	3	Yes	Yes	NA	GNSS (SPI Elite System GPSports)	5	14.4	19.8	
Guard et al. [142]	10 Male 18 Scottish youth Tier 3	4v4 + GK 5v5 6v4	J	45 × 34 39 × 39 23 × 23	1.32 1 1	153 142 53	4 × 4	2	Yes No No	NA	2, 3, free	GNSS + MEMS (MinimaxX, Catapult)	5 + 100	14.4	17.3	23.4
Guard et al. [143]	12 Male 18 Scottish youth Tier 3	4v4 + GK 6v4	J	40 × 30 45 × 34 49 × 37 23 × 23	1.33 1.32 1.32 1	120 153 181.3 53	4 × 4 4 × 4 4 × 4 8 × 2	2 2 2 1.5	Yes Yes Yes No	NA	2, 3, free	GNSS + MEMS (MinimaxX, Catapult)	5 + 100		21	24
Halouani et al. [187]	16 Male 18.3 Tunisian youth Tier 3	4v4	No	25 × 20	1.25	65.2	4 × 4	2	Yes & No	Yes	Free	GNSS (Playertek, Catapult)	10	13	18	
Hauer et al. [188]	17 Male 17.2 Austrian Tier 3	4v4 + GK	No	40 × 32	1.25	128	4 × 2	2	Yes	No	Free	LPM (Inmotiotech)	40	13	18	
Hodgson et al. [189]	8 Male 20 Tier 2	4v4 + GK	No	30 × 20 40 × 30 50 × 40	1.5 1.3 1.25	60 120 200	4 × 4	3	Yes	Yes	NA	GNSS + MEMS (MinimaxX, v.4.0, Catapult)	10 + 100		20.9	24.1
Ispirlidis [190]	22 Male 28.7 Greek Tier 3	10v10 + GK	No	75 × 65	1.15	221.6	4 × 8	NA	Yes	NA	Free	GNSS (Polar Team Pro)	5	14.4	19.8	25
Jastrzebski and Radziminski [191]	13 Male 27.1 Tier 3	4v4 + GK 5v5 + GK	No	40 × 30 43 × 33	1.3 1.3	120 118	4 × 4	3	Yes	Yes	Free	GNSS + MEMS (MinimaxX, v.4.0, Catapult)	10 + 100	13.8		23.7
Jastrzebski et al. [192]	13 Male 26.8 Tier 3 8 Female 22.5 Tier 3	4v4 + GK	No	40 × 30	1.3	120	4 × 4	3	Yes	Yes	Free	GNSS + MEMS (MinimaxX, v.4.0, Catapult)	10 + 100	14.4	19.8	25.2
Jastrzebski and Radziminski [193]	8 Male 27.5 Polish Tier 3 8 Female 19.1 Polish youth Tier 3	4v4 + GK	No	40 × 30	1.3	120	4 × 4	3	Yes	Yes	Free	GNSS + MEMS (MinimaxX, v.4.0, Catapult)	10 + 100	14.4	19.8	25.2
Köklu et al. [194]	15 Male 17 Turkish youth Tier 3	2v2 3v3 4v4	No	25 × 16 30 × 20 32 × 25	1.6 1.5 1.28	100	1 × 12 6 × 2 3 × 4 2 × 6	No 5 2 2	No	Yes	Free	GNSS (SPI Pro X, GPSports)	15	13	18	
Köklu et al. [195]	18 Male 18.2 Turkish youth Tier 3	3v3 4v4	No	30 × 20 32 × 25	1.5 1.28	100	4 × 4	2	No	Yes	Free	GNSS (SPI Pro X, GPSports)	15	14.4	19.8	
Langendam et al. [196]	10 Male 17.6 Dutch youth Tier 3 7 Male 18.7 Dutch Tier 2	4v4 + GK	No	60 × 40	1.5	240	4 × 4	2	Yes	NA	NA	GNSS + MEMS (Johan Sports)	10 + 100	14	20	
López-Fernández et al. [197]	16 Male 19.6 Spanish Tier 3	4v4	No	20 × 20 24.5 × 24.5 28.3 × 28.3	1	50 75 100	1 × 4	No	No	Yes	NA	GNSS (SPI Pro X, GPSports)	NA	13	18	
López-Fernández et al. [198]	21 Male NA Spanish youth Tier 3	3v3 4v4 5v5 6v6	No	30 × 20 32 × 25 37 × 27 30 × 40	1.5 1.33 1.37 0.75	100	4 × 4	4	No	NA	NA	GNSS (SPI Pro X, GPSports)	NA	13	18	
Lorenzo-Martínez et al. [199]	30 Male 24.2 Spanish Tier 2	4v4 + GK	No	30 × 35	0.85	105	4 × 4	2	Yes	NA	NA	GNSS + MEMS (Playertek, Catapult)	10 + 400	13	18	
Luchesi et al. [200]	16 Male NA Brazilian Tier 3	4v4 + GK	No	40 × 26 26 × 40	1.53 0.65	104	4 × 5	2	Yes	NA	NA	GNSS + MEMS (Polar Team Pro)	10 + 200		19.8	25
Madison et al. [141]	10 Male 23 Tier 2	3v3 4v4	No	20 × 15 40 × 25	1.3 1.6	50 125	6 × 4	1.5	Yes	NA	NA	GNSS + MEMS (Apex, StatSports)	18 + 100	13.4	17.8	22.3
Mallo and Navarro [201]	10 Male 18.4 Youth Tier 3	3v3 3v3 + GK	No	33 × 22	1.5	121 91	1 × 5	No	No	NA	NA	Optical	NA	13	18	
Mara et al. [202]	18 Female 24.3 Tier 3	4v4 5v5 6v6 7v7 8v8 9v9	No	40 × 40 50 × 40 60 × 40 70 × 40 80 × 68 90 × 68	1 1.25 1.5 1.75 1.18 1.32	200 200 200 200 340 340	2 × 5 2 × 5 2 × 7 2 × 7 2 × 9 2 × 9	2	Yes	NA	NA	GNSS (SPI Pro X, GPSports)	15	12.2	19.2	
Martín-García et al. [203]	21 Male 20.4 Spanish Tier 3	5v5 + GK 6v6 + 1 + GK 9v9 + GK	No J No	33 × 40 33 × 40 72 × 65	0.82 0.82 1.1	110 88 234	1 × 5 1 × 5 1 × 10	No	Yes	NA	NA	GNSS + MEMS (Viper Pod, StatSports)	10		19.8	25.2
Martín-García et al. [204]	25 Male 20.4 Spanish Tier 3	7v7 + 3 8v8 + 3	J	29 × 36 40 × 35	0.8 1.1	61 74	1 × 5	NA	Yes	NA	NA	GNSS + MEMS (Viper Pod, StatSports)	10		19.8	25.2
Modena et al. [205]	18 Male 28.7 Tier 2	4v4 3v3 + GK	No	30 × 20 40 × 30 30 × 20 40 × 30	1.5 1.3 1.5 1.3	75 150 75 150	4 × 4	3	Yes	Yes	NA	GNSS (Viper Pod, StatSports)	10		19.8	25.2
Nunes et al. [206]	23 NA 22.3 Tier 2	4v4	No	30 × 25	1.2	93.8	4 × 4	4	No	No	Free	GNSS + MEMS (ZEPP Play Soccer system)	NA	18		
Nunes et al. [207]	18 Male 21 Youth Tier 2	4v4	No	20 × 15 25 × 20 30 × 25	1.3 1.3 1.2	37.5 62.5 93.8	4 × 4	4	No	No	Free	GNSS + MEMS (ZEPP Play Soccer system)	NA		18	
Nunes et al. [206]	20 Male 22.3 Tier 2	4v2 4v3 4v4 5v4 6v4	J	20 × 15 25 × 20 30 × 25	1.3 1.25 1.2	50–125 42.8– 107 37.5–93.7 33.3–83.3 30–75	4 × 4	4	No	No	Free	GNSS + MEMS (ZEPP Play Soccer system)	NA		18	
Nunes et al. [208]	18 Male 21 NA	4v2 4v4 6v4	J No J	30 × 25	1.2	187.5 93.8 75	4 × 4	4	No	No	Free	GNSS + MEMS (ZEPP Play Soccer system)	NA		18	
Olthof et al. [209]	43 Male 17.9 Dutch youth Tier 3	4v4 + GK	No	40 × 30 68 × 47	1.3 1.4	120 319.6	5 × 4	4	Yes	Yes	Free	LPM (Inmotiotech)	42–100		19.8	
Owen et al. [21]	10 Male 27.6 European Tier 3	3v3 + GK 4v4 + GK 5v5 + GK 6v6 + GK 7v7 + GK 8v8 + GK 9v9 + GK	No	30 × 25 46 × 40 50 × 44 54 × 45 60 × 50 70 × 56 80 × 70	1.5 1.2 1.1 1.2 1.2 1.3 1.1	93.8 184 183.3 173.6 187.5 217.8 280	3 × 5	3	Yes	NA	Free	GNSS (MinimaxX, Catapult)	5	14.4	21.6	25.3
Owen et al. [210]	23 Male 25.3 European Tier 3	5v5	No	25 × 25	1	62.5	3 × 3	2	No	Yes	Free	GNSS + MEMS (Viper Pod, STATSports)	10 + 100		19.8	25.5
Papanikolaou et al. [211]	10 Male 21.7 NA	4v4 8v8	No	20 × 25 70 × 65	0.8 1.1	62.5 284.4	6 × 4 3 × 8	3 1.5	No	Yes	Free	GNSS + MEMS (Polar Team Pro)	10 + 200	14	21	
Praça et al. [212]	16 Male 20.1 Brazilian Tier 3	4v4 4v4 + GK	No	36 × 27	1.3	121.5 97.2	4 × 4	4	No Yes	NA	Free	GNSS (Polar Team Pro)	10	14.3	19.7	25.1
Rábano-Muñoz et al. [213]	10 Male 17.7 Spanish youth Tier 2 10 Male 24.1 Spanish Tier 2	4v4 + 2	J	40 × 30	1.3	120	4 × 4	2	No	Yes	Free	GNSS (SPI Elite System, GPSports)	5	14	18	
Rago et al. [214]	8 Male 23.6 Portuguese Tier 2	4v4 + GK	No	37 × 28	1.32	103.6	3 × 6	3	Yes	Yes	Free	GNSS + MEMS (SPI Pro X, GPSports)	15 + 100	13	16	22
Rebelo et al. [215]	18 Male 20.7 Tier 2	4v4 + GK 8v8 + GK	No	47.7 × 29.5 85.9 × 53.2	1.6	141 253.8	6 × 6 2 × 18	3	Yes	N	Free	GNSS + MEMS (SPI Elite System, GPSports)	5 + 100	13.1	16.1	19
Rago et al. 2018	14 Male 27.3 Italian Tier 3	7v7 + GK	No	45 × 35	1.28	98.4	1 × 10	No	Yes	NA	Free	GNSS (SPI Pro X, GPSports)	15	16	19	22
Rago et al. [56]	14 Male 27.6 Italian Tier 3	7v7 + GK	No	45 × 35	1.3	98.4	1 × 10	No	Yes	Yes	Free	GNSS + MEMS (SPI Elite System, GPSports)	5 + 100	16		
Rago et al. [56]	14 Male 27.6 Italian Tier 3	10v10 + GK	No	90 × 50	1.8	204.5	1 × 10	No	Yes	Yes	Free	GNSS + MEMS (SPI Pro X, GPSports)	15 + 100		16	22
Reinhardt et al. [216]	14 Male 23.5 German Tier 2	4v4 + GK	No	40 × 30	1.3	120	6 × 1.5	1.5	Yes	Yes	Free	GNSS (Polar Team Pro)	10	14.4	19.8	
Riboli et al. [131]	25 Male 27 Italian Tier 4	From 4v4 + GK to 10v10 + GK	No	From 30 × 20 to 105 × 65	From 0.8 to 1.6	From 60 to 426.5	4 × 4	4	Yes	Yes	Free	GNSS (K-Sport)	10	15	20	24
Riboli et al. [217]	25 Male 26 Italian Tier 4	From 3v3 + GK to 10v10 + GK From 3v3 to 10v10	No	From 20 × 30 to 95 × 40 From 30 × 20 to 105 x 65	From 0.5 to 1.5	From 43 to 569 From 67 to 341	NA	NA	Yes No	Yes	Free	GNSS (K-Sport)	10	15		24
Riboli et al. [218]	49 Male 17 Italian youth Tier 3 37 Male 18 Italian Youth Tier 3 58 Male 19 Italian youth Tier 3	From 3v3 + GK to 10v10 + GK with 1 or 2 additional players From 2v2 + GK to 10v10 + GK with 1 or 2 additional players From 3v3 + GK to 10v10 + GK with 1 or 2 additional players	NA	From 25 × 22 to 105 × 68 From 15 × 25 to 65 × 52 From 18 × 25 to 66 × 104	From 1 to 2.62 From 0.57 to 1.6 From 0.56 to 1.43	From 41.2 to 324.5 From 46.9 to 187.7 From 32 to 356.6	NA	NA	Yes	Yes	Free	GNSS (NA)	10	15	20	24
Rojas-Valverde et al. [219]	14 Male 20.9 Costa Rican Tier 2	7v7	No	30 × 20 40 × 30	1.5 1.3	42.9 85.7	2 × 10	3	Yes	Yes	Free	GNSS + MEMS (SPI Elite System, GPSports)	5 + 100	14.1	19.1	
Rowell et al. [133]	21 Male 25.2 Australian Tier 3	5v5 + GK	F	45 × 36	1.25	135	4 × 3	1	Yes	NA	Free	GNSS + MEMS (OptimeEye S5, Catapult)	10 + 100	15.1		
San Román-Quintana et al. [220]	14 Male 24.4 Spanish Tier 2	7v7 + GK	No	60 × 49	1.2	183.8	1 × 12	No	Yes	Yes	1, 2, 3	GNSS + MEMS (MinimaxX, v.4.0, Catapult)	10 + 100	13	18	21
Sanchez-Sanchez et al. [221]	12 Male 17.2 Youth Tier 2	4v4 + GK	No	40 × 30	1.3	120	3 × 8–10	4–6	Yes	Yes	Free	GNSS (K-Sport)	10	13		
Sannicandro et al. [222]	14 Male 24.7 Tier 3	5v5 + GK + 6 6v6 + GK + 6 7v7 + GK + 7	F	60 × 35	1.7	175 150 131.3	4 × 3	1	Yes	No	Free	GNSS (GPEXE^®^ System, Exelio)	18.18	14.5	19.9	25.2
Sannicandro et al. [223]	10 Male 24.7 Tier 3	5v5 + GK 5v5 + GK + 6	No F	60 × 35	1.7	175	6 × 3	1	Yes	No	Free	GNSS (GPEXE^®^ System, Exelio)	18.18	14.5	19.8	25.2
Sannicandro et al. [224]	20 NA 23.6 Tier 3	4v4 + GK	No	55 × 49 60 × 54	1.12 1.11	269.5 324	4 × 5	2	Yes	Yes	3	GNSS (GPEXE^®^ System, Exelio)	18.18	14.5	19.8	25.2
Santos et al. [225]	8 Male 20.1 Portuguese Tier 3	4v4	No	24 × 16 30 × 20 36 × 24	1.5	48 75 108	1 × 3	3	No	NA	NA	GNSS (Wimu Pro, Realtrack Systems)	NA	12	18	24
Santos et al. [226]	10 Male 20.1 Portuguese Tier 3	4v4 4v4 + GK	No	24 × 16 30 × 20 36 × 24 30 × 20 36 × 24	1.5	48 75 108 60 86.4	1 × 3	3	No Yes	Yes	NA	GNSS (Wimu Pro, Realtrack Systems)	10	12	18	24
Sparkers et al. [227]	16 Male 21 English Tier 3	4v4 + GK	No	29 × 24	1.2	69.6	6 × 7	2	Yes	NA	Free	GNSS + MEMS (OptimeEye X4, Catapult)	10 + 100		19.8	
Sparkers et al. [228]	12 Male 21 Tier 2	4v4 + GK	No	29 × 24	1.2	69.6	6 × 7	2	Yes	NA	Free	GNSS + MEMS (OptimeEye S5, Catapult)	10 + 100	14.4	19.8	
Stevens et al. [11]	33 Male 21 Dutch Tier 3 33 Male 17 Dutch youth Tier 3 62 Male 26 Dutch Tier 2 16 Female 24 Dutch Tier 3	5v5 + GK	No	40 × 34	1.2	113.3	4 × 7	2	Yes	Yes	Free	LPM (Inmotiotech)	24	14.4		
Vázquez et al. [138]	9 Male 26.2 Spanish Tier 3	6v6 + 1 6v6 + 1 + GK 6v6 + 1 + GK	J	20 × 30 25 × 40 50 × 40	0.7 0.62 1.25	46 66.7 133	4 × 5	2	No Yes	Yes	2	GNSS (SPI, GPSports)	1	13	18	21
Younesi et al. [53]	12 Male 28.1 Qatari Tier 3	3v3 3v3 + GK 4v4 4v4 + GK 6v6 6v6 + GK	No	20 × 27 20 × 27 22 × 32 22 × 32 28 × 40 28 × 40	0.74 0.74 0.68 0.68 0.7 0.7	90 67.5 90 54 90 38.6	3 × 3 3 × 3 3 × 4 3 × 4 3 × 6 3 × 6	2	No Yes No Yes No Yes	Yes	Free 3 Free 3 Free 3	GNSS (VX Sport)	10	14.4	19.8	

*ApP* area per player, *F* floaters, *GK* goalkeeper, *GNSS* global positioning system, *HSR* high-speed running, *J* jolly, *LPM* local position measurement, *L:W Ratio* length to width ratio, *MEMS* micro-electromechanical system, *min* minutes, *n* sample size, *NA* not available, *RCT* randomized controlled trial, *SR* sprint running, *VHSR* very high-speed running

### Main Models

**Fig. 2:**
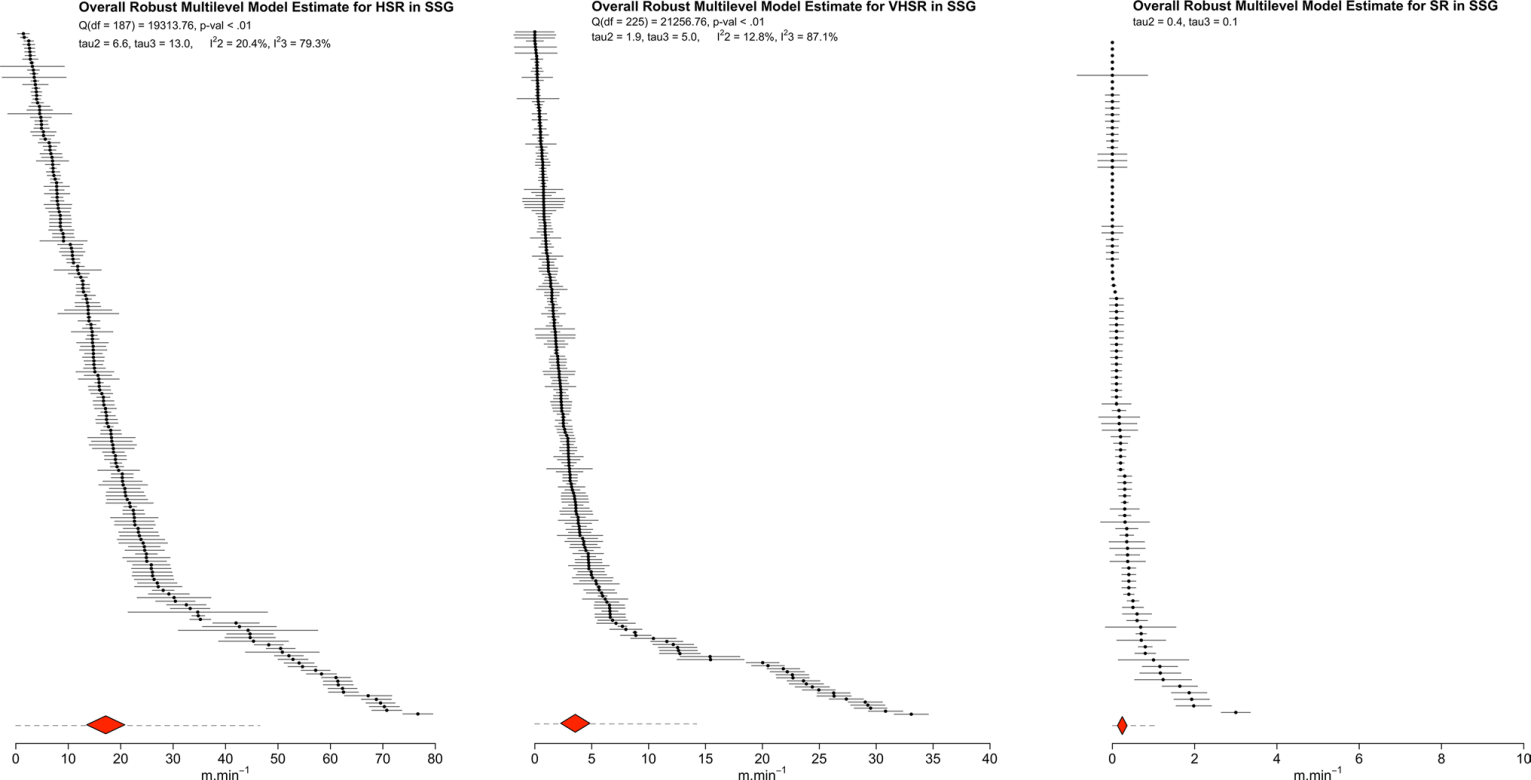
Ordered caterpillar plot presenting all effect sizes and 95% interval estimates from all included studies in small-sided games (SSG) meta-analyses. The *red polygon* presents the overall effect estimate and 95% confidence intervals, while the *dashed lines* present 95% prediction intervals. *HSR* high-speed running, *SR* sprint running, *VHSR* very high-speed running

**Fig. 3:**
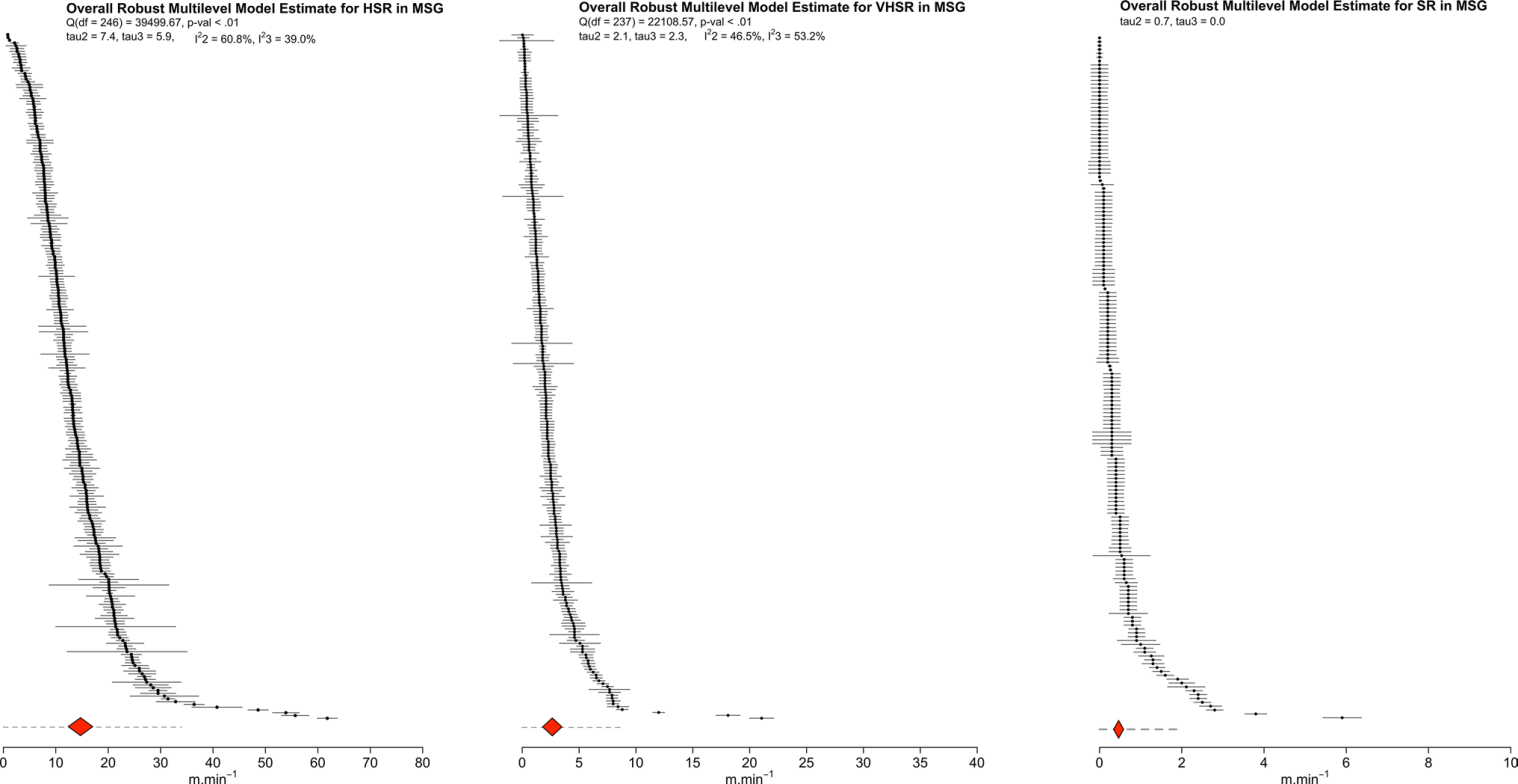
Ordered caterpillar plot presenting all effect sizes and 95% interval estimates from all included studies in medium-sided games (MSG) meta-analyses (high-speed running [HSR], very high-speed running [VHSR] and sprint running [SR]). The *red polygon* presents the overall effect estimate and 95% confidence intervals, while the *dashed lines* present 95% prediction intervals

**Fig. 4:**
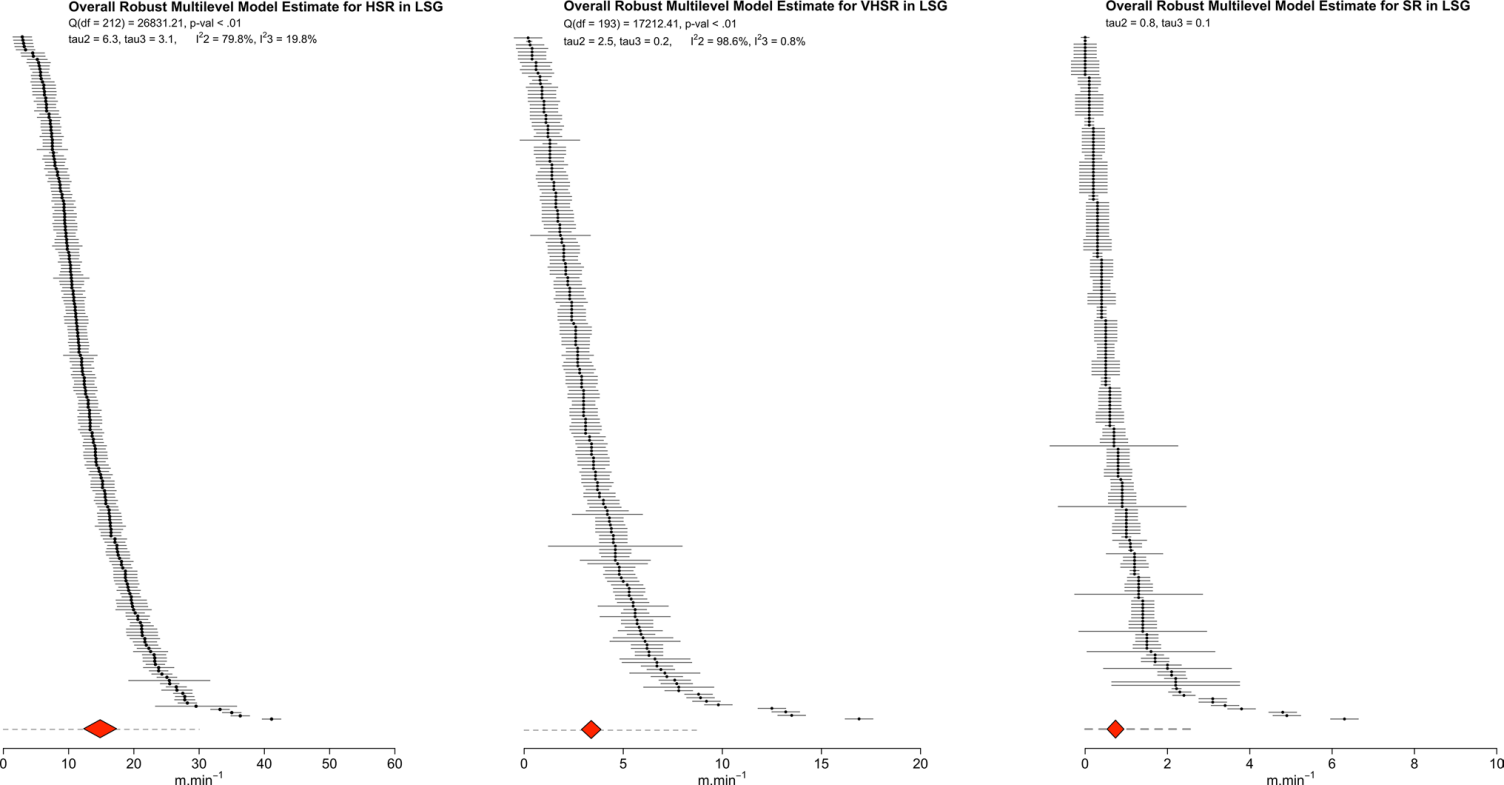
Ordered caterpillar plot presenting all effect sizes and 95% interval estimates from all included studies in large-sided games (LSG) meta-analyses. The *red polygon* presents the overall effect estimate and 95% confidence intervals, while the *dashed lines* present 95% prediction intervals. *HSR* high-speed running, *SR* sprint running, *VHSR* very high-speed running

**Fig. 5:**
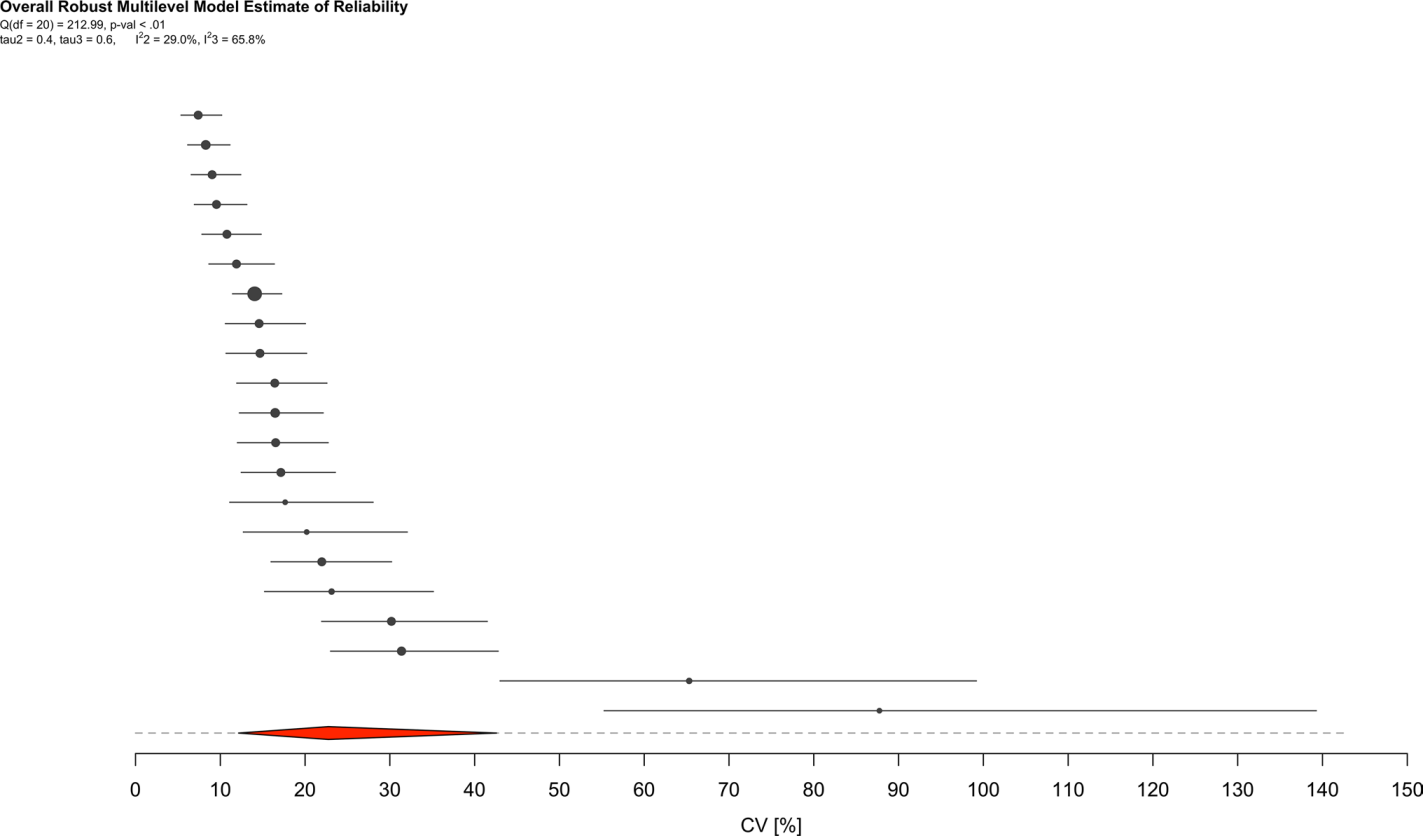
Ordered caterpillar plot presenting all effect sizes and 95% interval estimates from all included studies in intra-individual reliability meta-analysis. The *red polygon* presents the overall effect estimate and 95% confidence intervals, while the *dashed lines* present 95% prediction intervals

**Fig. 6:**
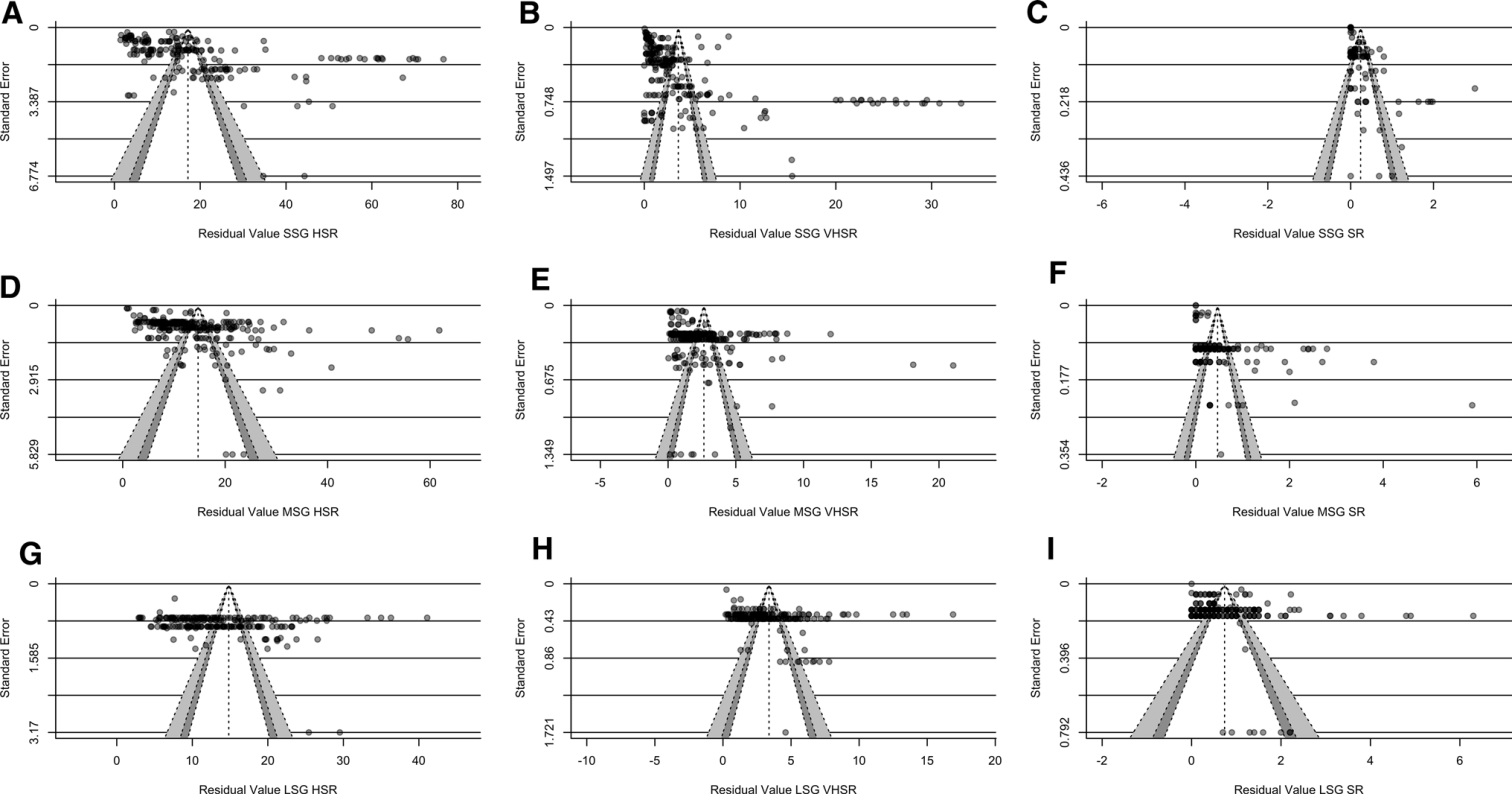
Funnel plots for risk of bias across studies with confidence levels of 90% (*white*), 95% (*dark gray*), and 99% (*light gray*): **A** small-sided games high-speed running (SSG HSR); **B** SSG VHSR; **C** SSG sprint running (SR); **D** medium-sided games (MSG) HSR; **E** MSG very high-speed running (VHSR); **F** MSG SR; **G** large-sided games (LSG) HSR; **H** LSG VHSR; and LSG SR. Egger’s regression test: **A** *F* test = 8.01, *p* < 0.01; **B** *F* test = 28.86, *p* < 0.001; **C** *F* test = 30.31, *p* < 0.001; **D** *F* test = 10.17, *p* < 0.01; **E** *F* test = 8.89, *p* < 0.01; **F** *F* test = 110.36, *p* < 0.001; **G** *F* test = 7.94, *p* = 0.01; **H** *F* test = 3.58, *p* = 0.08; and **I** *F* test = 1.41, *p* = 0.26

Table 4 and Figs. 2, 3, 4 and 5 present the number of clusters and estimates, the weighted point estimates with 95% CI, and the predictive point estimates with 95% PI for each meta-analysis. Asymmetrical scatter was evident in seven (Panels A–G) of the nine examined datasets (Fig. 6). Notably, to help with interpreting the results of our meta-analysis, we developed a companion web application, “Sided-games Training App”, which we suggest using to intuitively visualize the main findings of the meta-analytical and meta-regression models as well as to predict the expected high-speed, very high-speed, and sprint running exposure scenarios when planning soccer sided-games training (link to App: https://antonio-dello-iacono.shinyapps.io/Sided-games-Training-App/?_ga=2.181926951.1296146234.1647352519-774762236.1645808783).

**Table 4:** Summary of the main meta-analyses results

Sided-game format	External load measure	Number of		Pooled effects		
		Clusters	Estimates per cluster [median; range]	Estimate (m·min^−1^)	95% CI (lower to upper)	95% PI (lower to upper)
SSG	HSR	59	188 [2; 1–21]	17.2	13.5–20.8	0.0–46.5
	VHSR	65	226 [2; 1–21]	3.6	2.3–4.9	0.0–14.2
	SR	28	103 [2; 1–21]	0.2	0.1–0.4	0.0–1.1
MSG	HSR	45	247 [2; 1–59]	14.7	12.4–17.1	0.0–34
	VHSR	41	238 [2; 1–59]	2.6	1.8–3.5	0.0–9.0
	SR	17	177 [2; 1–59]	0.5	0.3–0.6	0.0–2.0
LSG	HSR	16	213 [2; 1–80]	14.8	12.3–17.4	0.0–30.0
	VHSR	17	194 [2; 1–80]	3.4	2.9–3.9	0.0–8.7
	SR	11	203 [2; 1–80]	0.7	0.5–0.9	0.0–2.6

*CI* confidence interval, *HSR* high-speed running, *LSG* large sided-games, *min* minute, *MSG* medium sided-games, *PI* prediction interval, *SR* sprint running, *SSG* small sided-games, *VHSR* very high-speed running

#### Small-Sided Games

The main models including all estimates of high-speed, very high-speed, and sprint running suggest that during SSG players are exposed, on average, to high-speed, very high-speed, and sprint distances with a weighted point and interval estimate of 17.2 m $\cdot$ min^−1^ (95% CI 13.5–20.8), 3.6 m $\cdot$ min^−1^ (95% CI 2.3–4.8), and 0.2 m $\cdot$ min^−1^ (95% CI 0.1–0.4), respectively. There was however noteworthy heterogeneity for all models (high-speed distance: *Q*_(187)_ = 19,313.75, *τ*_2_ = $\pm$ 6.6 [95% CI 5.8–7.5] and *τ*_3_ = $\pm$ 13.1 [95% CI 10.7–16.1], $I_{2}^{2}$ = 20.4% and $I_{3}^{2}$ = 79.3%; very high-speed distance: *Q*_(225)_ = 21,256.76, *τ*_2_ = $\pm$ 1.9 [95% CI 1.7–2.1] and *τ*_3_ = $\pm$ 5 [95% CI 4.2–6.0], $I_{2}^{2}$ = 12.8% and $I_{3}^{2}$ = 87.1%; sprint distance: *τ*_2_ = $\pm$ 0.4 [95% CI 0.3–0.5] and *τ*_3_ = $\pm$ 0.1 [95% CI 0.0–0.3]). The width of the 95% PI suggested that exposure could fall anywhere in the range of 0–46.5, 0–14.2, and 0–1.1 m $\cdot$ min^− 1^ for high speed, very high-speed, and sprint running distances, respectively.

#### Medium-Sided Games

The main models including all estimates of high-speed, very high-speed, and sprint running suggest that during MSG players are exposed, on average, to high-speed, very high-speed, and sprint distances with a weighted point and interval estimate of 14.7 m $\cdot$ min^−1^ (95% CI 12.4–17.1), 2.7 m $\cdot$ min^−1^ (95% CI 1.8–3.5), and 0.5 m $\cdot$ min^−1^ (95% CI 0.3–0.6), respectively. There was however noteworthy heterogeneity for all models (high-speed: *Q*_(246)_ = 39,499.67, *τ*_2_ = $\pm$ 7.4 [95% CI 6.7–8.2] and *τ*_3_ = $\pm$ 5.9 [95% CI 3.9–8.5], $I_{2}^{2}$ = 60.8% and $I_{3}^{2}$ = 39.0%; very high-speed distance: *Q*_(237)_ = 22,108.57, *τ*_2_ = $\pm$ 2.1 [95% CI 1.9–2.4] and *τ*_3_ = $\pm$ 2.3 [95% CI 1.4–3.3], $I_{2}^{2}$ = 46.5% and $I_{3}^{2}$ = 53.4%; sprint distance: *τ*_2_ = $\pm$ 0.7 [95% CI 0.6–0.8] and *τ*_3_ = 0.0 [95% CI 0.0–0.5]). The width of the 95% PI suggested that exposure could fall anywhere in the range of 0–34, 0–9.0, and 0–2 m $\cdot$ min^−1^ for high speed, very high-speed, and sprint running distances, respectively.

### Large-Sided Games

The main models including all estimates of high-speed, very high-speed, and sprint running suggest that during LSG players are exposed, on average, to high-speed, very high-speed, and sprint distances with a weighted point and interval estimate of 14.8 m $\cdot$ min^−1^ (95% CI 12.3–17.4), 3.4 m $\cdot$ min^−1^ (95% CI 2.9–3.9), and 0.7 m $\cdot$ min^−1^ (95% CI 0.5–0.9), respectively. There was however noteworthy heterogeneity for all models (high-speed: *Q*_(212)_ = 26,831.21, *τ*_2_ = $\pm$ 6.3 [95% CI 5.7–7.0] and *τ*_3_ = $\pm$ 3.1 [95% CI 0.0–7.0], $I_{2}^{2}$ = 79.8% and $I_{3}^{2}$= 19.8%; very high-speed distance: *Q*_(193)_ = 17,212.41, *τ*_2_ = $\pm$ 2.5 [95% CI 2.3–2.8] and *τ*_3_ =$\pm$ 0.2 [95% CI 0.0–1.6], $I_{2}^{2}$ = 98.6% and $I_{3}^{2}$ = 0.8%; sprint distance: *τ*_2_ = $\pm$ 0.84 [95% CI 0.8–0.9] and *τ*_3_ = $\pm$ 0.1 [95% CI 0.0–0.5]). The width of the 95% PI suggested the exposure could fall anywhere in the range of 0–30 m $\cdot$ min^−1^, 0–8.7 m $\cdot$ min^−1^, and 0–2.6 m $\cdot$ min^−1^for high speed, very high-speed, and sprint running distances, respectively.

### Intra-Individual Reliability

The meta-analysis of all intra-individual reliability estimates (21 across 7 clusters [median 2, range 1–12 estimates per cluster]) determined weighted and predictive point estimates with respective CI and PI equal to 22.8% (95% CI 12.2–42.6) and 22.7% (95% PI 3.6–143.1). There was however noteworthy heterogeneity with *Q*_(20)_ = 212.99, *τ*_2_ = $\pm$ 0.4 (95% CI 0.3–0.6) and *τ*_3_ = $\pm$ 0.6 (95% CI 0.2–1.4), $I_{2}^{2}$ = 29.0%, and $I_{3}^{2}$ = 65.8%.

### Meta-Regression Analyses

Table 5 displays the weighted point estimates with 95% CI for each moderator assessed in the meta-regression analyses.

**Table 5:** Summary of the meta-regression analyses

Sided-game format	External load measure	Moderating effects		Intercept (95% CI)
				Slope (95% CI)
SSG	HSR	Baseline (intercept)		15.6 m $\cdot$ min^−1^ (10.5 to 20.6)
		Speed thresholds	+ 1 km $\cdot$ h^−1^	–2.5 (–4.0 to –1.1)
		Area per player	+ 25 m^2^	2.5 (2.0 to 3.0)
		Length:width ratio	+ 0.2 (AU)	0.1 (–0.8 to 1.1)
		Game orientation	Score oriented	–1.3 (–5.2 to 2.6)
SSG	VHSR	Baseline (intercept)		2.2 m $\cdot$ min^−1^ (0.7 to 3.**7**)
		Speed thresholds	+ 1 km $\cdot$ h^−1^	–1.4 (–2.0 to –0.7)
		Area per player	+ 25 m^2^	0.6 (0.4 to 0.8)
		Length:width ratio	+ 0.2 (AU)	0.2 (–0.1 to 0.4)
		Game orientation	Score oriented	–0.2 (–1.3 to 0.8)
SSG	SR	Baseline (intercept)		1.0 m $\cdot$ min^−1^ (0.4 to 1.6)
		Speed thresholds	+ 1 km $\cdot$ h^−1^	–0.4 (–0.7 to –0.1)
		Area per player	+ 25 m^2^	0.1 (0.1 to 0.2)
		Length:width ratio	+ 0.2 (AU)	0.0 (–0.0 to 0.1)
		Game orientation	Score oriented	–0.0 (–0.2 to 0.1)
MSG	HSR	Baseline (intercept)		7.8 m $\cdot$ min^−1^ (3.4 to 12.2)
		Speed thresholds	+ 1 km $\cdot$ h^−1^	–1.6 (–4.0 to 0.8)
		Area per player	+ 25 m^2^	2.8 (2.1 to 3.4)
		Length:width ratio	+ 0.2 (AU)	0.5 (0.1 to 0.8)
		Game orientation	Score oriented	4.8 (0.2 to 9.5)
MSG	VHSR	Baseline (intercept)		0.7 m $\cdot$ min^−1^ (–0.3 to 1.6)
		Speed thresholds	+ 1 km $\cdot$ h^−1^	–0.8 (–1.2 to –0.4)
		Area per player	+ 25 m^2^	0.9 (0.7 to 1.1)
		Length:width ratio	+ 0.2 (AU)	0.3 (0.1 to 0.5)
		Game orientation	Score oriented	1.0 (0.6 to 1.4)
MSG	SR	Baseline (intercept)		0.3 m $\cdot$ min^−1^ (–1.0 to 1.5)
		Speed thresholds	+ 1 km $\cdot$ h^−1^	–0.1 (–0.7 to 0.5)
		Area per player	+ 25 m^2^	0.2 (0.1 to 0.4)
		Length:width ratio	+ 0.2 (AU)	0.0 (–0.2 to 0.2)
		Game orientation	Score oriented	0.3 (–0.3 to 0.9)
LSG	HSR	Baseline (intercept)		5.2 m $\cdot$ min^−1^ (–1.5 to 12.0)
		Speed thresholds	+ 1 km $\cdot$ h^−1^	–4.1 (–6.2 to –2.1)
		Area per player	+ 25 m^2^	1.9 (1.0 to 2.8)
		Length:width ratio	+ 0.2 (AU)	–0.3 (–1.2 to 0.7)
		Game orientation	Score oriented	7.4 (4.3 to 10.4)
LSG	VHSR	Baseline (intercept)		1.3 m $\cdot$ min^−1^ (–1.0 to 3.6)
		Speed thresholds	+ 1 km $\cdot$ h^−1^	–1.4 (–2.0 to –0.8)
		Area per player	+ 25 m^2^	0.8 (0.5 to 1.1)
		Length:width ratio	+ 0.2 (AU)	–0.2 (–0.5 to 0.1)
		Game orientation	Score oriented	0.3 (–2.5 to 3.1)
LSG	SR	Baseline (intercept)		0.7 m $\cdot$ min^−1^ (–0.6 to 2.0)
		Speed thresholds	+ 1 km $\cdot$ h^−1^	–0.3 (–0.9 to 0.3)
		Area per player	+ 25 m^2^	0.3 (0.1 to 0.4)
		Length:width ratio	+ 0.2 (AU)	–0.1 (–0.3 to 0.1)
		Game orientation	Score oriented	0.2 (–0.1 to 0.6)

*AU* arbitrary unit, *CI* confidence interval, *HSR* high-speed running, *LSG* large sided-games, *min* minute, *MSG* medium sided-games, *SR* sprint running, *SSG* small sided-games, *VHSR* very high-speed running

#### Velocity Thresholds

Meta-regression suggested that high-speed, very high-speed, and sprint running exposure were moderated by the velocity thresholds set to collect these external load measures. Specifically, per every unit increment or decrement ($\pm$ 1 km $\cdot$ h^−1^) from the anchored velocity references, high-speed running exposure changed, on average, 2.5 m $\cdot$ min^−1^ (95% CI 1.1–4.0), 1.6 m $\cdot$ min^−1^ (95% CI − 0.8 to 4.0), and 4.1 m $\cdot$ min^−1^ (95% CI 2.1–6.2) in SSG, MSG, and LSG, respectively. Similarly, very high-speed running exposure changed, on average, 1.4 m $\cdot$ min^−1^ (95% CI 0.7–2.0), 0.8 m $\cdot$ min^−1^ (95% CI 0.4–1.2), and 1.4 m $\cdot$ min^−1^ (95% CI 0.8–2.0) in SSG, MSG, and LSG, respectively. Finally, sprint running exposure changed, on average, 0.4 m $\cdot$ min^−1^ (95% CI 0.1–0.7), − 0.1 m $\cdot$ min^−1^ (95% CI − 0.7 to 0.5), and 0.3 m $\cdot$ min^−1^ (95% CI − 0.9 to 0.3) in SSG, MSG, and LSG, respectively.

#### Area per Player

The meta-regression suggested that high-speed, very high-speed, and sprint running exposure were moderated by the area per player consistently across all sided-game formats. Specifically, for every 25-m^2^ increment of the relative area per player from the anchored reference of 100 m^2^ per player, high-speed running exposure increased, on average, by 2.5 m $\cdot$ min^−1^ (95% CI 2.0–3.0), 2.8 m $\cdot$ min^−1^ (95% CI 2.1–3.4), and 1.9 m $\cdot$ min^−1^ (95% CI 1.0–2.8) in SSG, MSG, and LSG, respectively. Similarly, very high-speed running exposure increased, on average, by 0.6 m $\cdot$ min^−1^ (95% CI 0.4–0.8), 0.9 m $\cdot$ min^−1^ (95% CI 0.7–1.1), and 0.8 m $\cdot$ min^−1^ (95% CI 0.5–1.1) in SSG, MSG, and LSG, respectively. Finally, sprint running exposure increased, on average, by 0.1 m $\cdot$ min^−1^ (95% CI 0.1–0.2), 0.2 m $\cdot$ min^−1^ (95% CI 0.1–0.4), and 0.3 m $\cdot$ min^−1^ (95% CI 0.1–0.4) during SSG, MSG, and LSG, respectively.

#### Length:Width Ratio

The meta-regression suggested that the length:width ratio moderated high-speed, very high-speed, and sprint running exposure differently across the sided-game formats. In SSG, an increase was observed for high-speed (0.1 m $\cdot$ min^−1^ [95% CI − 0.8 to 1.1]) and very high-speed (0.2 m $\cdot$ min^−1^ [95% CI − 0.1 to 0.4]) but not in sprint (0.0 m $\cdot$ min^−1^ [95% CI − 0.0 to 0.1]) running exposure. Similarly, also in MSG, exposure to high-speed and very high-speed running increased, on average, by 0.5 m $\cdot$ min^−1^ (95% CI 0.1– 0.8) and 0.3 m $\cdot$ min^−1^ (95% CI 0.1–0.5), respectively, while no effects were found for sprint running (0.0 m $\cdot$ min^−1^ [95% CI − 0.2 to 0.2]). Contrasting moderating effects were observed in LSG, with decreases in high-speed (− 0.3 m $\cdot$ min^−1^ [95% CI − 1.2 to 0.7]), very high-speed (− 0.2 m $\cdot$ min^−1^ [95% CI − 0.5 to 0.1]), and sprint running exposure (− 0.1 m $\cdot$ min^−1^ [95% CI − 0.3 to 0.1]).

#### Game Orientation

The meta-regression suggested that high-speed, very high-speed, and sprint running exposure were moderated by the game orientation differently across the sided-game formats. In SSG, a decrease was observed for high-speed (− 1.3 m $\cdot$ min^−1^ [95% CI − 5.2 to 2.6]), very high-speed (− 0.2 m $\cdot$ min^−1^ [95% CI − 1.3 to 0.8]), and sprint (− 0.0 m $\cdot$ min^−1^ [95% CI − 0.2 to 0.1]) running exposure when the game was score oriented and included either goalkeepers or small goals. Contrasting moderation effects were observed in MSG, whereby exposure to high-speed, very high-speed, and sprint running, increased, on average, by 4.8 m $\cdot$ min^−1^ (95% CI 0.2–9.5), 1.0 m $\cdot$ min^−1^ (95% CI 0.6–1.4), and 0.3 m $\cdot$ min^−1^ (95% CI − 0.3 to 0.9), respectively, in the presence of goalkeepers or small goals. Similarly, game orientation also moderated LSG high-speed, very high-speed, and sprint running exposure with, on average, an increased exposure of 7.4 m $\cdot$ min^−1^ (95% CI 4.3–10.4), 0.3 m $\cdot$ min^−1^ (95% CI − 2.5 to 3.1), and 0.2 m $\cdot$ min^−1^ (95% CI − 0.1 to 0.6), respectively.

### Risk of Bias

**Fig. 7:**
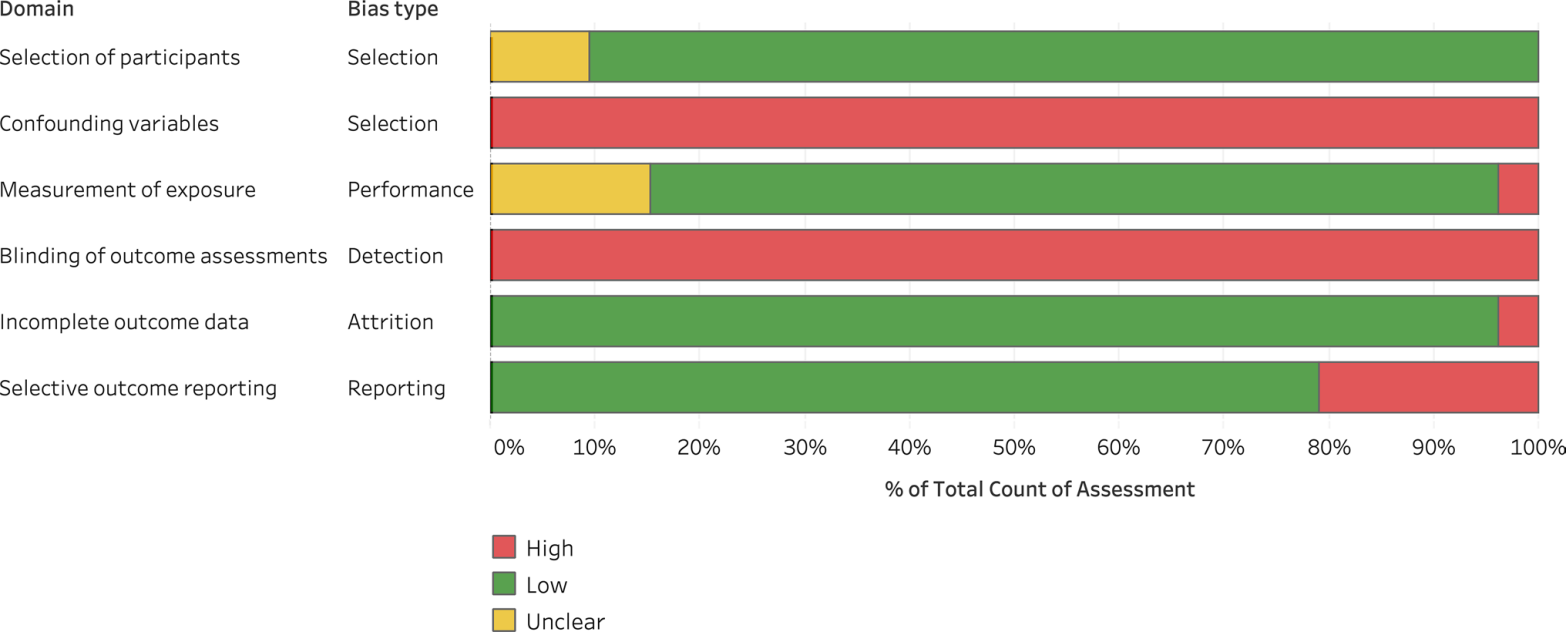
Assessment of risk of bias of studies included in the meta-analysis

Full results and summary of the RoBANS assessment of the included studies are presented in the ESM (https://osf.io/rf48s) and Fig. 7, respectively. Across all studies, the greatest risk of bias (100%) was observed in the confounding variables domain considering that none of the studies (*n* = 105) reported the dwell time required above the minimal velocity thresholds for locomotive actions to be recorded as high-speed very high-speed or sprinting effort, and most studies did not report the number of satellites obtained (*n* = 101) or the horizontal dilution of precision (*n* = 102). Similarly, a high risk of bias (100%) was observed in the domain pertaining to the blinding of outcome assessments as none of the studies (*n* = 105) reported any procedures adopted to blind the outcomes of the sided-games training. Risk of bias (20%) was also observed in the selective outcome reporting domain as 21 studies did not report descriptive statistics (i.e., mean, standard deviation and CI) of the external load outcomes. The lowest risk of bias (8.5%) was observed in the selection of participants, as only in 9 of the 105 studies the sample characteristics were not clearly reported.

## Discussion

Our systematic review and meta-analysis are the first to provide an exploratory summary and a quantitative synthesis of high-speed, very high-speed, and sprint running exposure and intra-individual reliability in soccer sided games from 104 and 7 studies, respectively. The main findings from our analysis were that high-speed, very high-speed, and sprint running exposure induced by sided games, irrespective of format, are not comparable to the corresponding outcomes reported for regular 11-a-side soccer matches. Moreover, poor reliability of these external load measures was found in SSG and MSG formats, suggesting that exposure is highly variable in sided games. Across sided-games formats, high-speed, very high-speed, and sprint running exposure were influenced by the tracking device velocity thresholds and playing constraints such as the relative area per player, pitch length-to-width ratio, and game orientation.

### High-Speed, Very High-Speed, and Sprint Running Exposure

**Fig. 8:**
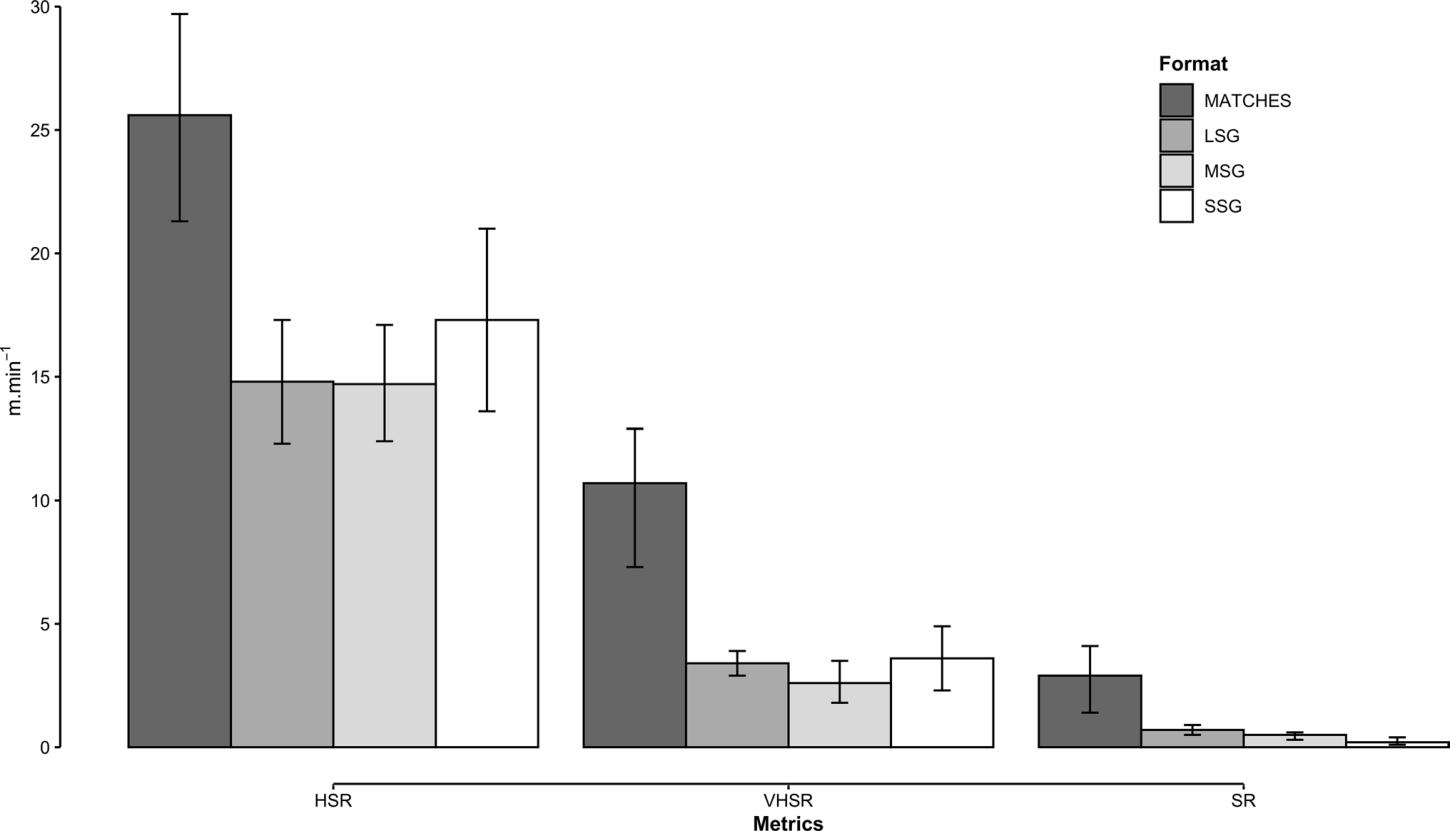
Comparative visualization of high-speed, very high-speed, and sprint running exposure between official matches and sided games. Data are presented as means and 95% confidence intervals

The systematic monitoring of external loads is core for the comprehensive evaluation of dose exposure during training and competition and the subsequent optimal planning and management of the training processes [229]. The main findings of this review provides insight for the use of sided games as integrated soccer-specific training [230–232], as a physical conditioning method [233] as well as for training load exposure strategies [232, 234, 235]. Promoting evidence-informed practices in soccer, the results of our meta-analysis confirm that sided games are inappropriate to replicate match play demands. To contextualize, across all sided-games formats, the pooled estimates were considerably lower than the analogous external load measures reported for official matches at the amateur level [236], in professional European competitions such as the English Premier League [45, 46], the Spanish La Liga [237], the Italian Serie A [238, 239], the French Ligue [240], and the German Bundesliga [241], in addition to the Union of European Football Associations Champions League [67, 68] and international tournaments of the Fédération Internationale de Football Association [69, 242]. For example, during regular 11-a-side soccer matches in competitions involving adult (i.e., age $\ge$ 17 years) soccer players of any sex and level, relative high-speed, very high-speed, and sprint running exposure ranges were 20.2–29.7 m $\cdot$ min^−1^, 7.1–12.8 m $\cdot$ min^−1^, and 1.3–3.9 m $\cdot$ min^−1^, respectively. Noticeably, the corresponding (i.e., same velocity thresholds collected with the same tracking technologies) pooled estimates (Table 4) from studies included in this meta-analysis were up to approximately six-fold lower (i.e., for high-speed, very high-speed, and sprint exposure, respectively: $\downarrow$ 9.9%, $\downarrow$ 83.4%, and $\downarrow$ 584% in SSG; $\downarrow$ 43.9%, $\downarrow$ 174%, and $\downarrow$ 182% in MSG; $\downarrow$ 38.4%, $\downarrow$ 111.3%, and $\downarrow$ 78% in LSG; Fig. 8). The evidence that sided games fail to fully replicate the high-speed demands of regular play [41, 44, 171, 243] has practical implications as described below.

From a tactical perspective, the evolution of elite soccer match play requires players to perform more high-speed and sprint actions to fulfill tactical responsibilities, whilst in and out of possession, and during ball possession transitions [45, 46]. These locomotor activities are also key determinants for successful performance [47] as high-speed and especially straight sprint running have been identified as the most frequent locomotive actions preceding goal situations, performed by either the scoring player and the assisting player [48, 49]. At a granular level, position-specific profiles have been reported with special reference to high-speed movement patterns particularly when contextualized with technical skills and tactical actions [244, 245]. In this regard, while tactical drills appear to provide the greatest combined physical, technical, and tactical training stimulus and transfer, it is plausible that sided games lack effectiveness to fully account for the multi-dimensional domains of the positional demands. The multi-positional drill nature and the reduced pitch sizes and player numbers characterizing sided games largely affect individual and collective tactical behavior [246] as smaller pitches (i.e., 88–145 m^2^ per player) and low player numbers (i.e. SSG and MSG) result in shorter inter-player distances [247], increased unpredictable short-distance movements [248], and greater movement variability in players’ pitch zones [249] compared with regular match play. Conversely, larger pitches (i.e., $>$ 216 m^2^ per player) with greater player numbers (i.e., LSG) lead to more regular positioning and reduce player movement variability, but at the expense of a smaller radius of free movement over longer distances [209, 249]. These considerations indicate that although sided-games training is appropriate to induce changes in collective behavior aimed at improving or refining tactical proficiency at the team level, it may not be fully effective to closely replicate the multi-dimensional positional patterns of match play with reference to high-speed movements, which is crucial when preparing players for the positional tactical demands of the modern game.

From a physical conditioning perspective, our study provides robust evidence for an informed planning of sided-games training in soccer. On the one hand, sided-games cannot be endorsed as a comprehensive method especially if the main training goal is to overload high-speed, very high-speed, and sprint running exposure. For example, assuming the pooled estimates from this meta-analysis, a typical sided-games training session, which usually consists of $\sim$ 15 min of effective playing time (see ESM; https://osf.io/5hzve), would be expected to induce, on average, total high-speed, very high-speed, and sprint running exposure of $\sim$ 235, $\sim$ 49, and $\sim$ 7 m, respectively. Comparisons with the corresponding relative outcomes for full matches (i.e., $\sim$ 375, $\sim$ 150, and $\sim$ 40 m, respectively) [45, 46, 67–69, 236–239, 241, 242] clearly highlight that sided games do not induce a sufficient overload stimulus for high-speed and sprint running exposure. In practical terms, such dose exposure and the underpinning physiological, biochemical, and neuromuscular responses may still contribute to maintain fitness in soccer players during the in-season period when sided games are implemented systematically through multiple weekly sessions as different formats but combined with other forms of training [250, 251]. However, the effectiveness of sided-games training alone to enhance high-speed and sprint running capabilities [3] or to compensate for the lack of match-induced exposure among non-starting players is questionable [252, 253]. Similarly, although some sided-games formats (i.e., SSG) may elicit mechanical loads due to repeated accelerations and decelerations to a level that is at least equivalent with peak periods of official match play [43], their effectiveness as longitudinal training interventions aimed at enhancing strength, jumping, and change of direction capabilities in soccer players is minimal [3, 254]. On the other hand, coaches and practitioners may use sided games to ensure progressive high-speed running exposure during the pre-season period when a gradual overload may be required as well as in-season to target a minimal dose exposure in tapering weeks and days or during congested fixture periods [47, 240].

Planning high-speed and sprint running training receives particular attention among soccer coaches and practitioners as optimal exposure strategies may also have a preventive role against injuries for which inadequate training dose is considered as a modifiable risk factor [235]. Unaccustomed volumes and spikes in sprint and near-to-maximal speed distances during competitive match-play have been reported to have harmful association with muscle injury occurrence [255, 256]; therefore, exposing soccer players to progressive and optimal sprint running doses may provide a preventive effect, especially for non-contact hamstrings injuries [257, 258]. This likelihood of muscle injuries is reasonably increased among non-starting players owing to the lack of match-induced high-speed and sprint running exposure, especially if these are not adequately compensated for during the training micro-cycle. Implementing training strategies with a particular focus on the ability to repeat and tolerate near-to-maximal and sprint actions [232, 259] would therefore appear relevant to the context of muscle injury preventive strategies. Furthermore, considering that most of the hamstrings injuries in soccer players occur because of altered running kinematics during maximal sprint actions [256, 260, 261], especially peaking at the latter stages of soccer match play [255, 262], specific drills that replicate the neuromuscular, mechanical, and physiological demands of sprint running may help refine the running technique and develop muscular stress resilience and tolerance resulting in indirect injury prevention benefits [263, 264]. With these programming subtleties in mind, the use of sided-games training as part of preventive strategies against hamstring injury through appropriate maximal speed exposure is questionable. First, only trivial sprint running distances (e.g., 5–12 m for a typical sessions lasting 15 min) can be covered during sided games unless very extensive training volumes and formats including small numbers of players (i.e., SSG) and very high relative areas per player ($>$ 300 m^2^ per player) are used [38, 131], which is rather impractical in the context of a full squad environment. Moreover, another critical reason is the likely lack of sprint specificity during sided games characterized by frequent short-distance (5–10 m) acceleration-like sprint movements as opposed to longer ($>$ 15 m) and near-to-maximal speed actions common in regular match play [47]. Arguably, the different sprint-specific locomotive profiles between sided games and matches require distinct hamstrings recruitment and activity at the hip and knee joints, which could limit the potential benefits of specific strengthening transfer and the protective role against hamstrings injuries occurring during sprint running [265, 266].

### Intra-Individual Reliability

Quantifying the repeatability of the external load demands during sided games and drawing inferences about the associated individual responses and adaptations are paramount for the design of soccer training programs [229, 251]. In this meta-analysis, high-speed and sprint running exposure measures showed poor reliability with CV values that ranged from 12.2 to 42.6%. Notably, while separate pooled estimates could have not been computed for each speed category because of the small number of estimates, an exploratory inspection (see ESM) of the intra-individual reliability values emphasizes that the external load variables most associated with fatigue and muscle damage in soccer [233] present the lowest consistency, with distances covered at very high speed and sprinting showing CV values ranges of 8–62.4% and 16.1–19.1%, respectively. These findings were expected given that the locomotive demands in sided games are random and uncontrolled [246, 248, 249]. Practically, this may have important implications for training load management and monitoring as the PIs of the CV of our meta-analysis (Fig. 5, 3.6–143.1%) reveal that sided-games training can overexpose some players as well as underexpose others with respect to the individual dose exposure sought by the coaching or sports science staff [39]. Accordingly, conditioning methods complementing sided games or designed intentionally as isolated high-speed and sprint drills or soccer-specific circuits may be beneficial if the training session aim is to expose players to these demands with a low degree of uncertainty. It is also noteworthy that the pooled estimates of the variability reported above encapsulate different sources of variability whose precise partition could not be determined [267, 268]. To explain, an estimate of intra-individual variability extracted from each individual study is a mean estimate of the sample in the study. As such, the pooled estimates in our meta-analysis likely captured: (1) technical variability from each study due to the monitoring devices and experimenters; (2) day-to-day variability in studies that implemented a test–retest design with between-day repeated measurements; (3) variability in response to the same sided-games training between individuals; and (4) true intra-individual variability or individual variation in response to the same training. While the magnitude of some sources of variability (i.e., technical variability) may be extracted from the literature [269, 270], other sources of variability (e.g., day-to-day biological variability, variability in response to the same training, and true intra-individual variability) are specific to the studied population and may require studies including randomized repeated interventions and reliability trials to be quantified. This is impractical in studies conducted in highly ecological environments. Moreover, the evidence on the intra-individual variability of the external load measures during sided-games training is weak (i.e., *n* = 7 studies) and pertinent only to SSG and MSG formats. While future research studies should purposefully address this topic to expand the knowledge available to date, it is advisable for coaching or sports science staff to account for intra-individual reliability in their load prescription and management strategies [251]. In fact, understanding the underpinning sources of intra-individual variability may help in interpreting training responses more accurately both at the group and individual level [267, 268]. For example, intra-individual variability provides information that allows inferences about whether inter-individual responses differences are true or a simple artefact of intra-individual variation. When evaluating inter-individual response differences, it is imperative to discern between the systematic or true response and intra-individual variation (e.g., day-to-day biological variability, variability in response to the same training, and true intra-individual variability). In some circumstances, the intra-individual variation may be large enough to contain a large proportion if not all inter-individual differences are apparent in training responses. Therefore, inter-individual comparisons based on average response values and failing to account for intra-individual variability may lead to biased conclusions. Similarly, intra-individual variation is also paramount when evaluating response differences at the individual level. In fact, accounting for intra-individual variability may facilitate inference as to whether true response differences occurred or should be attributed to concurrent training dependent factors (i.e., other training stimuli from the same training session) or to alternative factors independent from training (i.e., biological day-to-day variability). In this case, comparing a single response observation with a rolling baseline (i.e., average of several previous responses) that incorporates individual compatibility or equivalence intervals accounting for intra-individual variability is a viable option [271].

### Effects of the Between-Study Heterogeneity

In designing this systematic review and meta-analysis, our foremost research question was: *“What high-speed and sprint running exposure and associated reliability to expect by implementing sided-games training in soccer?”* Thereafter, and building upon the main findings of the meta-analysis, we aimed to provide a robust analysis of the magnitude of high-speed and sprint running exposure and the influence of common programming variables to facilitate informed training prescription, periodization, and load management planning strategies. To this end, taking into account the high risk of bias observed in some of the RoBANS domains and the uncertainty around the pooled estimates because of the large between-study heterogeneity in addition to the recommendations of Cochrane on matters regarding the number of studies included in meta-analyses [272] and the presence of asymmetry observed in the funnel plots [273], we calculated and recommend considering the 95% PIs reported in Table 4. In the context of this meta-analysis, the 95% PIs describe the range of effects to expect in 95% of future similar studies involving random samples of soccer players whom we intend to expose to high-speed and sprint running by implementing sided-games training. As expected, the 95% PIs were wider than the 95% CIs across all pooled estimates, confirming that the variation around external loads in sided-games training is multifactorial and influenced by several factors such as training variables, playing constraints, individual characteristics, or simply noise due to measurement error and biological variation. While a comprehensive investigation of all potential sources of between-study heterogeneity was computationally and practically unfeasible (e.g., limited number of estimates per factor and missing data), in the next section, we address the main potential sources of heterogeneity and interpret the related practical implications [64].

### Effects of Moderators

In this section, besides addressing and explaining the heterogeneity influencing the pooled estimates, we provide several practical suggestions for coaches and practitioners aiming to use different sided-games formats and to manipulate playing constraints for high-speed and sprint running exposure-focused training planning and prescription. To this end, we recommend using the “Sided-games Training App” and the “Planner” tab to simulate expected exposure scenarios unfolding from alternative sided-games training manipulations.

The finding that all pooled estimates across all sided-games formats were moderated and changed as a factor of the velocity thresholds is logical. Simply, lower and higher cut-off values set as velocity thresholds in the monitoring devices directly offset the magnitudes of external load measures toward greater and smaller outcomes, respectively. In view of the wide scale and the considerable variability found in the literature regarding the definitions of high-speed, very high-speed, and sprint running and corresponding velocity thresholds (Table 3), we suggest our meta-regression results as a practical programming tool (Table 5). Here, practitioners, sport scientists, and researchers may consider the parameters of the moderating effects to adjust the expected high-speed and sprint running exposures when using velocity thresholds that deviate from the anchored values that we used in our meta-analysis models. The simplicity of using a correcting factor is immediately advantageous for training prescription and load monitoring purposes as well as likely beneficial to facilitate data sharing and knowledge exchange between sport science departments and research groups [274].

Unimodal moderating effects on pooled estimates across all sided-games formats were found for the area per player variable, suggesting that high-speed and sprint running exposure can be progressively increased by implementing sided games with larger playing areas or lower player density. This robust finding encapsulates evidence showing that increased pitch sizes lead to greater inter-player and inter-team distances, resulting in larger spaces available to reach high-speed and near-to-maximal speed running [3, 38, 131, 209]. While previous studies recommended using sided-games formats with relative areas of 180–200 m^2^ $\cdot$ player, 200–300 m^2^ $\cdot$ player, and > 320 m^2^ $\cdot$ player to replicate the external load demands of regular matches [38, 131, 218], our main and meta-regression analyses provide highly powered results and robust evidence. Specifically, we suggest designing sided games, irrespective of the format characteristics, with relative playing areas approximately respectively equal to 200 m^2^ $\cdot$ player, 325 m^2^ $\cdot$ player, and > 365 m^2^ $\cdot$ player to induce relative high-speed, very high-speed, and sprint running exposure comparable to matches’ outcomes. As illustrated above for the threshold velocity, the anchored reference point for the area per player variable (100 m^2^) and the parameters of the moderating effects can be used as practical and useful tools when designing and planning sided-games sessions selectively targeting specific training goals [12, 15, 250].

Game orientation moderated high-speed and sprint running exposure differently across sided games, which appears to contradict the common belief and one of the conclusions from the recent umbrella review of Clemente et al. [3], supporting the notion that using goalkeepers and small scoring targets consistently reduces the external loads during sided-games training. Meta-regression suggested that score-oriented formats reduced high-speed and sprint running exposure in SSG with an opposite trend in both MSG and LSG. These contrasting results can be explained by a few technical tactical reasons and methodological pitfalls unfolding from studies where the comparative effects between sided games including the presence of goalkeepers or small goals and possession formats were investigated. From a tactical perspective, as elaborated above, the greater player and team dispersion characterizing MSG and LSG formats as well as the greater dimensions in larger pitch areas likely promote a more direct and vertical playing style with more frequent long-distance high-speed and sprint actions performed in and out of possession, and during ball possession transitions especially under exacerbating contextual constraints such as opponent pressure, score status, and reduced playing time [23, 149, 246, 275]. On the contrary, SSG formats with smaller pitch areas impose reduced positional dispersion and inter-player distances to preserve the spatial equilibrium on the field, and more importantly, to maintain or regain ball possession, which is a necessary condition for rapid goal scoring attempts [23, 186]. In this regard, greater frequencies of technical actions, among which shots to the opponent’s goal and shots far away from the opponent’s goal area, in particular, were reported in SSG compared with MSG and LSG formats [276–278]. This reasonably implies that fewer high-speed and sprint running actions are required to successfully score in small formats in consideration of the paired relationships between player positioning, score attempt actions, and external load variables [279]. Finally, most of the studies purposefully designed to investigate the comparative effects between score-oriented and possession-oriented SSG failed to adjust for the areas per player when goalkeepers were included, thus resulting in consistent smaller relative ratios. Therefore, the lower high-speed and sprint running exposure reported in score-oriented SSG formats is likely attributable to the moderating effects of the area per player as extensively explained above rather than due to the game orientation characteristics.

The conceptual and tactical considerations made about the moderating effects of the score-orientation constraint may, in part, also explain why the length:width ratio influences high-speed and sprint running exposure differently across sided-games formats. Mainly sided-games formats with equal length and width dimensions induce higher movement synchronization in both longitudinal and lateral directions, which facilitates a balanced dispersion of the players across the entire playing area, thereby resulting in an elongated playing shape style with a higher likelihood of increased distances covered at high speed [23, 246]. It is not entirely clear why an opposite moderating trend was found in LSG, with high-speed and sprint running exposure progressively decreasing as a factor of higher length:width ratios. However, it is plausible that the interaction between large player numbers ($\ge$ 8 vs 8) and a stretched pitch shape in the longitudinal direction may confine teams’ dispersion, particularly in response to transition play, thus causing a reduction in the effective playing space especially in the lateral corridors and diagonally, which ultimately limits the chances to perform high-speed actions [23, 246]. To summarize, while higher length:width ratios may increase high-speed and very high-speed running exposure during SSG and MSG, a balanced ratio should be maintained in LSG for the same purposes.

## Limitations

In conducting this systematic review and meta-analysis, we have identified a few limitations that warrant consideration. First and foremost, this meta-analysis included studies for which research designs and protocols were not pre-registered and pre-scrutinized (e.g., SPIRIT) according to strict standards suggested for observational studies (e.g., STROBE) or randomized controlled trials (e.g., CONSORT) [274]. However, this is a common and unavoidable limitation in meta-analysis studies when synthesizing training exposure investigated in applied settings and under highly ecological conditions, thus lacking proper internal validity. Given that gray literature searches make important contributions to systematic reviews as their exclusion can lead to exaggerated estimates of intervention effectiveness [280, 281], our decision not to undertake a gray literature search could be regarded as a limitation. Quantifying intervention effectiveness, however, was not our research objective as we were interested in the synthesis and quantification of sided-game high-speed, very high-speed, and sprint running exposure rather than the effectiveness of sided games as a fitness intervention. We also had concerns relating to the absence of peer review and that the inclusion of unpublished data can itself introduce bias as any studies located may be an unrepresentative sample of all unpublished studies [88], and, as in other fields, unpublished studies represent a very small proportion of included studies and rarely impact the results and conclusions of a review [282]. We acknowledge a single‑language bias, given that we included only studies reported in English; again, however, non-English studies represent a very small proportion of studies (in this instance, *n* = 2) and therefore have little impact on a review’s conclusions [282]. The overall pooled sample included mostly male adult soccer players and only 66 female participants, thus whether the main findings can be confidently generalized to female populations or to youth soccer players require further research. The grouping of high-speed, very high-speed, and sprint distance outputs between different tracking technologies has inherent notable flaws owing to the variety of devices, tracking approaches, sampling rates, filtering methods, and data-processing algorithms [274]. Finally, the relatively low number of estimates per dataset pertaining to sided-games characteristics such as the presence and type of coach encouragement, number of touches, position-specific data, and tactical instructions restricted any examination of the associated moderating effects on exposure to high-speed, very high-speed, and sprint running during sided-games training. On a similar note, while the overall number of estimates of intra-individual reliability from SSG and MSG formats was sufficient to conduct a meta-analysis, we could not extend the main findings to LSG formats or address and explain any potential sources of heterogeneity.

## Conclusions

Our study is the first to provide a quantitative synthesis of high-speed, very high-speed, and sprint running exposure and associated intra-individual reliability during soccer sided-games. We found that high-speed, very high-speed, and sprint running exposure during sided-games training is much lower than in official matches as well as showing poor reliability, irrespective of the sided-games formats. Coaches and practitioners choosing to use sided games could consider manipulating playing constraints such as area per player, game orientation, and length:width ratio, and cross-checking the velocity thresholds set in the tracking devices when planning high-speed and sprint running exposure-focused training and monitoring. Further work is warranted through well-designed and unbiased studies to improve the understanding of the possible sources of heterogeneity observed for high-speed, very high-speed, and sprint running exposure and the variability around these external load measures.

## Declarations

### Availability of data and material

All data are available in the Open Science Framework by accessing: https://osf.io/a4xr2/files/.
